# An Efficient Population Density Method for Modeling Neural Networks with Synaptic Dynamics Manifesting Finite Relaxation Time and Short-Term Plasticity

**DOI:** 10.1523/ENEURO.0002-18.2018

**Published:** 2019-01-17

**Authors:** Chih-Hsu Huang, Chou-Ching K. Lin

**Affiliations:** 1 Department of Neurology, National Cheng Kung University Hospital, College of Medicine, National Cheng Kung University, Tainan, Taiwan 70403; 2 Innovation Centre of Medical Devices and Technology, National Cheng Kung University Hospital, College of Medicine, National Cheng Kung University, Tainan, Taiwan 70403

**Keywords:** colored noise, diffusion approximation, exponential integrate-and-fire model, Langevin equations

## Abstract

When incorporating more realistic synaptic dynamics, the computational efficiency of population density methods (PDMs) declines sharply due to the increase in the dimension of master equations. To avoid such a decline, we develop an efficient PDM, termed colored-synapse PDM (csPDM), in which the dimension of the master equations does not depend on the number of synapse-associated state variables in the underlying network model. Our goal is to allow the PDM to incorporate realistic synaptic dynamics that possesses not only finite relaxation time but also short-term plasticity (STP). The model equations of csPDM are derived based on the diffusion approximation on synaptic dynamics and probability density function methods for Langevin equations with colored noise. Numerical examples, given by simulations of the population dynamics of uncoupled exponential integrate-and-fire (EIF) neurons, show good agreement between the results of csPDM and Monte Carlo simulations (MCSs). Compared to the original full-dimensional PDM (fdPDM), the csPDM reveals more excellent computational efficiency because of the lower dimension of the master equations. In addition, it permits network dynamics to possess the short-term plastic characteristics inherited from plastic synapses. The novel csPDM has potential applicability to any spiking neuron models because of no assumptions on neuronal dynamics, and, more importantly, this is the first report of PDM to successfully encompass short-term facilitation/depression properties.

## Significance Statement

Our study successfully solve an outstanding problem, how to reduce the dimension of population density equations when realistic synaptic dynamics is incorporated. With the newly proposed Fokker–Planck formalism, population density method (PDM) is conferred short-term plasticity (STP) properties and therefore becomes more widely applicable. As such, our method offers an opportunity to use the PDM to gain new insights into neural mechanisms of brain functions that are strongly dependent on STP synapses. This is the first step toward macroscopic description of large-scale neural network activities, reflected in some commonly used neurophysiological measurements, e.g., EEG, MEG, fMRI, and voltage-sensitive dye imaging data.

## Introduction

The population density method (PDM) appears as a time-saving alternative to direct numerical simulations [i.e., Monte Carlo simulation (MCS)] of large-scale neural networks of spiking neurons (Knight et al., 1996; Brunel and Sergi, 1998; Brunel, 2000; Nykamp and Tranchina, 2000; Omurtag et al., 2000; Casti et al., 2002; Huertas and Smith, 2006; Augustin et al., 2013). In this method, biophysically similar neurons are grouped into a population, and a probability density function for each population is defined to describe the variability of state variables of such neurons. The evolution of this density function is governed by a master equation (generally a partial differential equation such as a Fokker–Planck equation). A prominent advantage of PDM is that if the master equation can be easily solved in analytical or numerical ways, precise estimates of the quantities of interest can be obtained much faster than MCS.

Because of the significant impact of synaptic dynamics on network dynamics, PDMs must ultimately include accurate descriptions of synaptic kinetics to be widely applicable. Unfortunately, incorporating more synaptic dynamics increases the dimension of density functions, leading to a catastrophic decline in computational efficiency because of the increasing difficulty of solving high-dimensional master equations. How to incorporate realistic synaptic dynamics in a computationally efficient manner therefore becomes an outstanding problem in the field of PDMs (Apfaltrer et al., 2006; Tranchina, 2009).

Although many previous studies addressed this issue (Haskell et al., 2001; Nykamp and Tranchina, 2001; Apfaltrer et al., 2006; Rangan and Cai, 2006; Ly and Tranchina, 2007; Rangan et al., 2008), they left out some unsolved problems (section 7.10 in Tranchina, 2009). Recently, Ly (2013) developed a principled dimension-reduction method, called modified mean-field method (MMFM), that could deal with those unsolved problems. Nevertheless, we found that the MMFM might not be a robust method and not suitable for nonlinear spiking neuron models. The previous studies concentrated, to the best of our knowledge, only on synaptic dynamics with finite decay and neglected another important synaptic attribute, the short-term plasticity (STP; Markram and Tsodyks, 1996; Markram et al., 1998; Zucker and Regehr, 2002) that also played an important role in certain specific network dynamics, such as reverberatory bursting activity in *in vitro* and *in vivo* neuronal networks (Volman et al., 2007; Gritsun et al., 2011), self-organized criticality (Levina et al., 2007), anticipative neural responses (Fung et al., 2012), and sustained population activity related to working memory (Barak and Tsodyks, 2007; Mongillo et al., 2008). Therefore, our goal in the present study is to develop a new solution that allows the PDM to include realistic synaptic dynamics of both finite decay and STP in a computationally efficient manner.

In this study, we present a novel and efficient PDM, called colored-synapse PDM (csPDM), to achieve our goal. The strategy behind the csPDM is also to take advantage of dimension-reduction methods, relying on the following scheme: (1) synaptic dynamics are assumed to operate in the diffusive limit so that the dynamic equations of neuronal dynamics are converted into Langevin equations with colored driving noise and all synapse-associated state variables are transformed into input terms (Moreno-Bote and Parga, 2004; Richardson and Gerstner, 2005; Destexhe and Rudolph-Lilith, 2012), and (2) the probability density function method for this dynamic system is then employed (Wang et al., 2013; Barajas-Solano and Tartakovsky, 2016). As such, the synapse-associated state variables are not viewed as the system state variables of the resulting Langevin equations, leading to the reduction of the dimension of density functions. As a consequence, incorporating realistic synaptic dynamics does not decrease the computational efficiency when adopting csPDM, because it does not increase the dimension of master equations.

We compare the simulated network activities from the csPDM with those from MCSs, original full-dimensional PDMs (fdPDMs), and MMFMs. The numerical results show that the results of the csPDM reveal good agreement with those of the MCS and fdPDM in both the steady-state and dynamic regimes. The csPDM provides more accurate simulations than the MMFM and remains more computationally efficient than the fdPDM. We hope that the csPDM will be valuable to computational neuroscientists who seek accurate and efficient network modeling tools to explore effects of synaptic dynamics with/without STP properties on macroscopic behaviors of neural networks.

## Materials and Methods

Here, we describe the equations and parameters used for neuronal, synaptic and network modeling. We present the model (master) equations of the fdPDM, csPDM, and MMFM. The corresponding closed-form solutions for evaluating firing rate responses (network activities) are also described. At the end of this section, we present the numerical method used to solve the model equations of the csPDM as well as the methods used to quantify simulation errors. A summary of the notation for all the main dynamical variables and physiological parameters is given in Table 1.

### Network models

The large-scale network considered here is a neuronal population of *N* uncoupled exponential integrate-and-fire (EIF) neurons (Fourcaud-Trocmé et al., 2003) receiving only synaptic inputs from the outside. We choose the EIF model mainly because it can offer better prediction of spiking times for a given current input than the leaky integrate-and-fire models and quadratic ones (Fourcaud-Trocmé et al., 2003). In addition, its adaption version, i.e., the adaptive EIF model (Brette and Gerstner, 2005), is capable of reproducing various firing patterns observed in *in vivo* recordings of cortical cells (Naud et al., 2008; Touboul and Brette, 2008). In fact, the csPDM derived in the present study can be directly applied to simulate neural networks of adaptive EIF neurons.

In this network, the external synaptic inputs to each neuron are assumed to be mediated by *m* different types of neurotransmitter receptors. For the *s*th type of receptors, there are presumably *c_s_* synaptic connections. In other words, each neuron has totally $\sum_{s=1}^{s=m}c_{s}$ external synaptic connections. The synaptic input on each connection is a set of unitary spiking events whose random arrival times are assumed to be governed by an inhomogeneous Poisson process. *ν_s_*(*t*) denotes the mean rate of the Poisson process for the input through the *s*th-type receptor. to satisfy basic premises of the PDM, two arbitrary synaptic inputs are assumed to be statistically independent if they are mediated by different types of receptors and to be statistically identical if mediated by the same type.

### Single neuron models

For each neuron, the dynamics of its membrane voltage, *V* (*t*), is described by

(1) $$C\frac{d}{dt}V=-g_{l}\left(V-E_{l}\right)+g_{l}\kappa e^{\frac{V-V_{T}}{\kappa }}+I_{\text{syn}}\left(V,t\right),$$

where $I_{\text{syn}}\left(V,t\right)$ is the total synaptic current generated by the external synaptic inputs, *C* is the membrane capacitance, *g_l_* is the leak conductance, *E_l_* is the leak reversal potential, *κ* is the sharpness factor, and *V_T_* is the threshold voltage. The spiking mechanism is the following: a spike is triggered at *t_s_* when *V* (*t_s_*) reaches a cutting voltage, *V_c_* (in general, $V_{c}\geq V_{T}$). Afterward, *V* is immediately reset to a resetting voltage, *V_r_*, and, then, clamped for a refractory period, *τ_ref_*.

### Synapse models

Specifically, $I_{\text{syn}}\left(V,t\right)$ is the sum of all postsynaptic currents mediated by various types of neurotransmitter receptors:

(2) $$I_{\text{syn}}\left(V,t\right)=\sum_{s=1}^{m}I_{s}\left(V,t\right)=-\sum_{s=1}^{m}g_{s}\left(t\right)\left(V-E_{s}\right),$$

where *g_s_*(*t*) is the (collective) synaptic conductance for the input mediated by the *s*th type of receptors, and *E_s_* is the corresponding synaptic reversal potential. Equation 2 refers to conductance-based synaptic models.

The synaptic dynamics means the dynamics of the *g_s_*(*t*). In our treatment, the synaptic conductance follows first order kinetics, where the rise in conductance at the arrival of a unitary synaptic event is instantaneous and the decline is exponential. Therefore, the dynamic equation for the synaptic conductance has the following form:

(3) $$\tau_{s}\frac{d}{dt}g_{s}=-g_{s}+\sum_{j}\Gamma_{s}\delta \left(t-t_{j}^{s}\right),$$

where *τ_s_* is the decay or synaptic time constant and Γ*_s_* is the total variation of the synaptic conductance induced by a single event. The size of the instantaneous jump in conductance equals to $\Gamma_{s}/\tau_{s}$. $t_{j}^{s}$ is the arrival time of the *j*th synaptic event, and $\sum_{j}\delta \left(t-t_{j}^{s}\right)$ refers to the spike train consisting of synaptic events summed from all synaptic connections through the *s*th-type receptor.

In our numerical examples, the value of Γ*_s_* is set according to the following equation:

(4) $$\Gamma_{s}=C\ln\left(1-\frac{\Delta V_{s}}{E_{s}-E_{l}}\right),$$

where Δ*V_s_* is a free parameter defined as the instant voltage modulation provoked by a single synaptic event when the membrane voltage is *E_l_* just before the event arrives in cases where *κ* (in Eq. 1) is in the limit of κ→0 and the synaptic dynamics has a zero-order kinetics, i.e., $\tau_{s}\to 0$.

### Short-term plastic synapses

What stated above does not consider STP property on synaptic connections. In the following, we concentrate on how to add it into the synaptic dynamics. A typical dynamic model of STP was the one proposed by Barak and Tsodyks (2007). They introduced two running parameters to describe the change of the connection weight of a synaptic coupling in a phenomenological way. Following the same idea, the Γ*_s_* in Equation 3 is replaced by

(5) $$\Gamma_{s}\to \Gamma_{s}u_{s}\left(t\right)x_{s}\left(t\right),$$

where the running parameters, *u_s_* and *x_s_*, are governed by

(6) $$\frac{d}{dt}u_{s}=\frac{U_{s}-u_{s}}{\tau_{s,f}}+\sum_{j}U_{s}\cdot \left(1-u_{s}\right)\delta \left(t-t_{j}^{s}\right),$$

(7) $$\frac{d}{dt}x_{s}=\frac{1-x_{s}}{\tau_{s,r}}-\sum_{j}x_{s}u_{s}\delta \left(t-t_{j}^{s}\right)$$

at the level of a single synapse. The jump size of the synaptic conductance caused by a spike now depends on *u_s_* and *x_s_*. *u_s_* is the running utilization parameter and *x_s_* is the running fraction of available neurotransmitters. The dynamic value ranges from *U_s_* to 1 for *u_s_* and from 0 to 1 for *x_s_*. *U_s_* refers to the base level of *u_s_*. *u_s_* = 1 means a presynaptic spike is allowed to use all available neurotransmitters, and *x_s_* = 1 means all neurotransmitters are available.

Briefly, the Equations 6, 7 depict a phenomenon, where when a synaptic spike arrives, *u_s_* increases by an amount of $U_{s}\cdot \left(1-u_{s}\right)$, and, afterward, decays to its baseline level, *U_s_*, with the facilitation time constant, *τ_s_*_,_*_f_*, and, meanwhile, *x_s_* decreases by an amount of *x_s_u_s_* and recovers to its baseline value of 1 with the recovery time constant, *τ_s_*_,_*_r_*, afterward. In general, *τ_s_*_,_*_f_* and *τ_s_*_,_*_r_* are short, ranging from hundreds of milliseconds to seconds (Markram and Tsodyks, 1996; Markram et al., 1998). Besides *τ_s_*, *E_s_*, and Γ*_s_*, each synaptic connection with the STP property is also characterized by three additional parameters: *U_s_* (initial utilization parameter) and *τ_s_*_,_*_f_* as well as *τ_s_*_,_*_r_* (facilitation and recovery time constants, respectively), which control the type of the STP property from strong depression ($\tau_{r}>\tau_{f}$ and relatively large values of *U_s_*) to strong facilitation ($\tau_{f}>\tau_{r}$ and small values of *U_s_*; Barak and Tsodyks, 2007).

Note that we keep the subscript *s* in the Equations 5–7 above, because it is likely that the synaptic connections through different types of transmitter receptors have different STP types.

### MCS

We are interested in the network activity, i.e., the output population firing rate, of such a neuronal population in response to a given set of $\nu_{s}\left(t\right)$ (s∈{1,2,…,m}) as a whole. This quantity can be computed by the direct use of the MCS, where states of all neurons and synapses are explicitly traced according to Equations 1–3 or Equations 1–7 if STP properties are involved. For all our simulation examples, MSC is performed with the brian2 simulator (Stimberg et al., 2014). In all simulations, the network composes of *N* = 10,000 EIF neurons, and *c_s_* = 200 for all receptor types.

In the MCS, the output population firing rate is calculated by

(8) $$r\left(t\right)=\frac{n_{s}\left(t,t+\Delta t\right)}{N\Delta t},$$

where $n_{s}\left(t,t+\Delta t\right)$ is the total number of spikes produced by all neurons in the population within the time period of (t,t+Δt), and Δt is a small time window. Δ *t* is always set to 1 ms in our simulation examples.

### fdPDM

The assumptions and conditions, under which the fdPDM provides an exact description of network activity, have been previously discussed (Nykamp and Tranchina, 2000; Omurtag et al., 2000).

Specifically, the fdPDM is a density-based modeling approach to directly estimate *r*(*t*) with a known distribution of states of neurons, which is described by a joint probability density function, $\rho \left(V,\tilde{g},t\right)$, defined by

(9) $$\int_{\Omega }\rho \left(V,\tilde{g},t\right)d\Omega =\mathrm{Pr}\left\{\left(V\left(t\right),\tilde{g}\left(t\right)\right)\in \Omega \right\},$$

where $\tilde{g}=\left[g_{1},g_{2},\ldots ,g_{m}\right]$ is the conductance state vector composed of *m* synaptic conductance variables. Ω is a subdomain within the state space bounded by the definition domains of all state variables. In general, the definition domain of $\left\{g_{s}|s\in \left\{1,2,\ldots ,m\right\}\right\}$ ranges from 0 to *∞*. About the membrane voltage *V*, its upper bound is *V_c_*, and its lower bound is given by $\min\left(E_{l},V_{r},E_{s}\right)|s\in \left\{1,2,\ldots ,m\right\}$. Equation 9 states that the integration of the density function over Ω is the probability of finding neurons whose states are within that subdomain in a large neuronal population. Note that, here, we do not consider STP properties on synaptic connections to illustrate our main ideas in the present study in a simple way. We will consider these properties only for the csPDM later.

Unlike in the MCS, where one has to track every neuron individually, in the fdPDM, one just track one density function for each population. As explained in details in previous papers (Nykamp and Tranchina, 2000; Haskell et al., 2001; Apfaltrer et al., 2006; Ly, 2013), the master equation governing the evolution of $\rho \left(V,\tilde{g},t\right)$ forms a (1+ *m*)-dimensional (in space) partial-integral differential equation:

(10) $$\frac{\partial }{\partial t}\rho \left(V,\tilde{g},t\right)=-\frac{\partial }{\partial V}J_{V}-\frac{\partial }{\partial \tilde{g}}{\tilde{J}}_{g},$$

where the voltage probability flux, i.e., the probability flux along the voltage-direction, *J_V,_* is given by

$$J_{V}\left(V,\tilde{g},t\right)=\frac{1}{C}\left[-g_{l}\left(V-E_{l}\right)+g_{l}\kappa e^{\frac{V-V_{T}}{\kappa }}-\sum_{s=1}^{m}g_{s}\left(V-E_{s}\right)\right]\rho \left(V,\tilde{g},t\right)$$

(11) $$=F_{V}\left(V,\tilde{g}\right)\rho \left(V,\tilde{g},t\right),$$

and ${\tilde{J}}_{g}=\left[J_{g_{1}},J_{g_{2}},\ldots ,J_{g_{m}}\right]$ is the conductance probability flux vector composed of *m* conductance probability fluxes. The conductance probability flux, $J_{g_{s}}$, is given by

(12) $$J_{g_{s}}\left(V,\tilde{g},t\right)=-\frac{g_{s}}{\tau_{s}}\rho \left(V,\tilde{g},t\right)+c_{s}\nu_{s}\left(t\right)\int_{g_{s}-\Gamma_{s}/\tau_{s}}^{g_{s}}\rho \left(V,g_{1},\ldots ,g_{s-1},g\prime ,g_{s+1},\ldots ,g_{m},t\right)dg\prime .$$

The population firing rate, *r*(*t*), is obtained by integrating the voltage probability flux across *V_c_* over conductance, yielding

(13) $$r\left(t\right)=\int_{\;\Omega_{g\prime }}J_{V}\left(V_{c},\overset{˜}{g},t\right)d\overset{˜}{g}.$$

$\Omega_{g\prime }$ denotes the subdomain where $J_{V}\left(V_{c},\tilde{g},t\right)>0$, meaning that neurons at *V_c_* intend to cross the boundary from below to generate spikes in this domain. As seen, for a given set of inputs, *ν_s_*(*t*) (s∈{1,2,…,m}), we need to solve the Equation 10 to obtain the output response of the neuronal population, i.e., the output population firing rate, *r*(*t*). For solving that equation, the boundary conditions are:

$$J_{V}\left(V_{r}^{+},\tilde{g},t\right)-J_{V}\left(V_{r}^{-},\tilde{g},t\right)=J_{V}\left(V_{c},\tilde{g},t-\tau_{ref}\right),$$

$$J_{V}\left(V_{lb},\tilde{g},t\right)=0,$$

$$J_{g_{s}}\left(V,g_{1},\ldots ,g_{s-1},0,g_{s+1},\ldots ,g_{m},t\right)=0,$$

$$J_{g_{s}}\left(V,g_{1},\ldots ,g_{s-1},\infty ,g_{s+1},\ldots ,g_{m},t\right)=0,$$

where *V_lb_* denotes the lower bound of the membrane voltage. The former boundary condition results from the resetting mechanism of spiking, meaning that the voltage probability flux across *V_c_*, accounting for the generation of action potentials, re-enters the state space on *V_r_* after the refractory period. The latter ones mean no probability fluxes flow outward the domain through other boundaries. As stated, the boundary conditions result in the conservation of the number of neurons. That is to say, the integration of the density function over the domain always equals to one in the absence of refractory period.

The computational time required for solving the master equation depends on the number of its dimensions. Generally, solving a high-dimensional master equation is highly time consuming. Previous studies (Apfaltrer et al., 2006; Tranchina, 2009) have suggested that the fdPDM cannot be considered as a time-saving alternative to the MCS if the dimension of the master equation exceeds three. In the following, two other methods used to tackle this issue are described. MMFM is a documented method, and csPDM is a new method proposed in this paper.

### MMFM

MMFM is proposed by Ly (2013) for speeding up the estimation of the population firing rate. In this method, $\tilde{g}$ in the Equation 10 is viewed as a deterministic parameter so that the master equation becomes a one-dimensional partial differential equation:

(14) $$\frac{\partial }{\partial t}\rho \left(V,\tilde{g},t\right)=-\frac{\partial }{\partial V}\left[F_{V}\left(V,\tilde{g}\right)\rho \left(V,\tilde{g},t\right)\right].$$

In this case, a steady-state firing rate for a given $\tilde{g}$ is obtained by using the mean-field model, given by

(15) $$\bar{r}\left(\tilde{g}\right)=\{\begin{array}{ll} 0 & \text{if}\;F_{V}\left(V_{T},\tilde{g}\right)\leq 0,\\ \frac{1}{\tau_{ref}+\int_{V_{lb}}^{V_{c}}\frac{1}{F_{V}\left(V,\tilde{g}\right)}dV} & \text{otherwise}. \end{array}$$

Recall that the synaptic inputs for different types of neurotransmitter receptors are assumed to be independent mutually; therefore, synaptic conductances for different receptor types are also independent with each other. Furthermore, they only depend on their individual inputs. So, it is realized that the value of *g_s_* evolves isolatedly. For a large neuronal population, one can define a marginal conductance density function, $\rho_{s}\left(g_{s},t\right)$, for *g_s_* by analogy with $\rho \left(V,\tilde{g},t\right)$. Also, the integration of $\rho_{s}\left(g_{s},t\right)$ over a certain space on *g_s_* is the probability of finding neurons whose values of *g_s_* are located in that space. According to what derived by Ly (2013), the master equation governing the time evolution of $\rho_{s}\left(g_{s},t\right)$ is yielded as

(16) $$\frac{\partial }{\partial t}\rho_{s}\left(g_{s},t\right)=-\frac{\partial }{\partial g_{s}}{\bar{J}}_{s}\left(g_{s},t\right),$$

where

(17) $${\bar{J}}_{s}\left(g_{s},t\right)=-\frac{g_{s}}{\tau_{s}}\rho_{s}+c_{s}\nu_{s}\left(t\right)\int_{g_{s}-\Gamma_{s}/\tau_{s}}^{g_{s}}\rho_{s}\left(g\prime ,t\right)dg\prime .$$

The boundary conditions for solving Equation 16 are ${\bar{J}}_{s}\left(0,t\right)={\bar{J}}_{s}\left(\infty ,t\right)=0$. Assuming that the values of all elements of $\tilde{g}$ are drawn from the marginal conductance density functions individually, MMFM estimates the time-varying firing rate, r(t) , with expected value of $\bar{r}\left(\tilde{g}\right)$ conditioned on $\tilde{g}$, that is,

(18) $$r\left(t\right)=\int \bar{r}\left(\tilde{g}\right)\prod_{s=1}^{m}\rho_{s}\left(g_{s},t\right)d\tilde{g}.$$

As such, instead of solving a (1 + *m*)-dimensional partial-integral differential equation in the fdPDM, one only needs to solve *m* one-dimensional partial differential equations simultaneously for estimating *r*(*t*) in the MMFM. Ly suggested that MMFM had higher computational efficiency than fdPDM, especially when the values of $\bar{r}$ at all possible points of $\tilde{g}$ could be computed before simulations.

### Colored-synapse population density method (csPDM)

This section presents the derivation of the csPDM. csPDM is inspired by the probability density method for Langevin equations with colored noise (Wang et al., 2013; Barajas-Solano and Tartakovsky, 2016). So, we start by presenting how to transform the Equation 1 into a stochastic Langevin equation through the diffusion approximation of *g_s_*.

In the diffusion limit, under the condition that the mean rate of synaptic input received by each neuron, $c_{s}\nu_{s}$, is sufficiently high and the jump size $\Gamma_{s}/\tau_{s}$ for each synaptic spike is small enough, the random synaptic conductance $g_{s}\left(t\right)$ can be treated as the Ornstein–Uhlenbeck process (Uhlenbeck and Ornstein, 1930; Risken, 1996), whose dynamics is given by (Richardson, 2004)

(19) $$\tau_{s}\frac{d}{dt}g_{s}=-g_{s}+\sum_{j}\Gamma_{s}\delta \left(t-t_{j}^{s}\right)\approx -g_{s}+\mu_{g_{s}}+\sqrt{2\tau_{s}}\sigma_{g_{s}}\xi \left(t\right),$$

where $\mu_{g_{s}}$ is the mean value of $g_{s}\left(t\right)$ and $\sigma_{g_{s}}$ is the standard deviation of $g_{s}\left(t\right)$. ξ(t) is a *δ*-correlated white-noise process of unit variance. Introducing a new variable ${\bar{g}}_{s}=\frac{1}{\sigma_{g_{s}}}\left(g_{s}-\mu_{g_{s}}\right)$ and substituting for *g_s_* in the equation above, we obtain a new Ornstein–Uhlenbeck process for ${\bar{g}}_{s}$:

(20) $$\tau_{s}\frac{d}{dt}{\bar{g}}_{s}=-{\bar{g}}_{s}+\sqrt{2\tau_{s}}\xi \left(t\right).$$

${\bar{g}}_{s}$ has zero mean and unit variance (Risken, 1996); and its autocorrelation function is

(21) $$C_{s}\left(\tau \right)=〈{\bar{g}}_{s}\left(0\right){\bar{g}}_{s}\left(\tau \right)〉=e^{-\frac{\tau }{\tau_{s}}}.$$

As a result, ${\bar{g}}_{s}$ is a colored noise due to the exponential form of $C_{s}\left(\tau \right)$ and finite synaptic time constant, i.e., $\tau_{s}>0$. Replacing $g_{s}\left(t\right)$ in the Equation 1 with $\mu_{g_{s}}+\sigma_{g_{s}}{\bar{g}}_{s}$, we obtain

(22) $$\frac{dV}{dt}=\frac{1}{C}\left[-g_{l}\left(V-E_{l}\right)+g_{l}\kappa e^{\frac{V-V_{T}}{\kappa }}-\overset{m}{\underset{s=1}{\sum }}\mu_{g_{s}}\left(V-E_{s}\right)\right]+\overset{m}{\underset{s=1}{\sum }}\frac{1}{C}\sigma_{g_{s}}\left(E_{s}-V\right){\overset{¯}{g}}_{s}\left(t\right)={ℋ}_{0}\left(V,{\overset{˜}{\mu }}_{g}\right)+{ℋ}_{1}\left(V,{\overset{¯}{\sigma }}_{g},\overset{˜}{\overset{¯}{g}}\left(t\right)\right)$$

as the new dynamic equation for the membrane voltage, where ${\overset{˜}{\mu }}_{g}=\left[\mu_{g_{1}},\mu_{g_{2}},\ldots ,\mu_{g_{m}}\right]$, ${\overset{˜}{\sigma }}_{g}=\left[\sigma_{g_{1}},\sigma_{g_{2}},\ldots ,\sigma_{g_{m}}\right]$, and $\overset{˜}{\overset{¯}{g}}=\left[{\overset{¯}{g}}_{1},{\overset{¯}{g}}_{2},\ldots ,{\overset{¯}{g}}_{m}\right]$. So, as shown in Equation 22, the fluctuation of V(t) now is characterized by a Langevin equation with *m* independent colored noises $\left\{{\bar{g}}_{s}|s\in \left\{1,2,\ldots ,m\right\}\right\}$ resulting from synaptic dynamics with finite synaptic time constants (Destexhe and Rudolph-Lilith, 2012). That is why we use “colored-synapse” as the prefix of this method.

Barajas-Solano and Tartakovsky (2016) proposed a closed-form quasi-Fokker–Planck equation as the master equation for Langevin equations driven by colored noise. They considered a dynamic system characterized by the stochastic Langevin equation in *n* dimensions

(23) $$\frac{d}{dt}x_{i}=v_{i}\left(x,t\right)={〈v_{i}\left(x,t\right)〉}_{0}+v_{i}\prime \left(x,t\right)$$

for i=1,2,…,n, where $x={\left[x_{1},x_{2},\ldots ,x_{n}\right]}^{T}$. Each $v_{i}\left(x,t\right)$ is decomposed into a deterministic function or “mean-field velocity” ${〈v_{i}\left(x,t\right)〉}_{0}$ and a stochastic fluctuation term $v_{i}\prime \left(x,t\right)$. The authors proposed a quasi-Fokker–Planck equation:

(24) $$\frac{\partial p}{\partial t}=-\nabla \cdot \left({〈v〉}_{0}p\right)+\nabla \cdot \left(D\nabla p\right)$$

as the master equation governing the temporal evolution of the joint probability density function of the system states, p(x,t). The stochastic diffusion tensor, **D**, is calculated by

(25) $$D=\underset{t\to \infty }{\lim}\int_{0}^{t}〈v\prime \left(0\right)v\prime^{T}\left(\tau \right)〉\exp\left[\tau J^{T}\left(x\right)\right]d\tau$$

if fluctuation velocity components are exponentially autocorrelated and mutually uncorrelated, i.e.,

$$〈v_{i}\prime \left(0\right)v_{i}\prime \left(\tau \right)〉=\sigma_{i}^{2}\exp\left(-\tau /\tau_{i}\right),$$

$$〈v_{i}\prime \left(0\right)v_{j}\prime \left(\tau \right)〉=0,\;i\neq j.$$

$J^{T}\left(x\right)$ is the Jacobian of the mean-field velocity with components $J_{ij}\left(x,t\right)=\partial {〈v_{i}\left(x,t\right)〉}_{0}/\partial x_{j}$. Considering *V*, ${ℋ}_{0}\left(V,{\overset{˜}{\mu }}_{g}\right)$, and ${ℋ}_{1}\left(V,{\overset{˜}{\sigma }}_{g},\overset{˜}{\overset{¯}{g}}\right)$ in Equation 22 as **x**, ${〈v_{i}\left(x,t\right)〉}_{0}$, and $v_{i}\prime \left(x,t\right)$ in Equation 23, respectively, we can yield the master equation for the marginal voltage density function, $\rho_{V}\left(V,t\right)$, by using Equation 24, as the following:

(26) $$\frac{\partial }{\partial t}\rho_{V}\left(V,t\right)=-\frac{\partial }{\partial V}\left[{ℋ}_{0}\left(V,{\overset{˜}{\mu }}_{g}\right)\rho_{V}\right]+\frac{\partial }{\partial V}\left[D\left(V,{\overset{˜}{\sigma }}_{g}\right)\frac{\partial }{\partial V}\rho_{V}\right]=-\frac{\partial }{\partial V}{\overset{¯}{J}}_{V}\left(V,{\overset{˜}{\mu }}_{g},{\overset{˜}{\sigma }}_{g},t\right),$$

where

(27) $${\bar{J}}_{V}\left(V,{\tilde{\mu }}_{g},{\tilde{\sigma }}_{g},t\right)={ℋ}_{0}\left(V,{\tilde{\mu }}_{g}\right)\rho_{V}-D\left(V,{\tilde{\sigma }}_{g}\right)\frac{\partial }{\partial V}\rho_{V}.$$

By using Equation 25, we obtain

$$D\left(V,{\tilde{\sigma }}_{g}\right)=\underset{t\to \infty }{\lim}\int_{0}^{t}〈{ℋ}_{1}\left(V,{\tilde{\sigma }}_{g},\tilde{\bar{g}}\left(0\right)\right){ℋ}_{1}\left(V,{\tilde{\sigma }}_{g},\tilde{\bar{g}}\left(\tau \right)\right)〉\exp\left[\tau \frac{\partial }{\partial V}{ℋ}_{0}\left(V,{\tilde{\mu }}_{g}\right)\right]d\tau$$

(28) $$=\sum_{s=1}^{m}{\left[\frac{\sigma_{g_{s}}\left(E_{s}-V\right)}{C}\right]}^{2}\cdot \underset{t\to \infty }{\lim}\int_{0}^{t}C_{s}\left(\tau \right)\exp\left(\frac{-\tau }{\tau_{\text{eff}}}\right)d\tau$$

$$=\sum_{s=1}^{m}{\left[\frac{\sigma_{g_{s}}\left(E_{s}-V\right)}{C}\right]}^{2}\cdot \underset{t\to \infty }{\lim}\int_{0}^{t}\exp\left(\frac{-\tau }{\tau_{s}}\right)\exp\left(\frac{-\tau }{\tau_{\text{eff}}}\right)d\tau$$

(29) $$=\sum_{s=1}^{m}{\left[\frac{\sigma_{g_{s}}\left(E_{s}-V\right)}{C}\right]}^{2}\cdot \frac{\tau_{s}\tau_{\text{eff}}}{\tau_{s}+\tau_{\text{eff}}}$$

where

(30) $$\tau_{\text{eff}}=\frac{C}{g_{l}-g_{l}e^{\frac{〈V〉-V_{T}}{\kappa }}+\sum_{s=1}^{m}\mu_{g_{s}}},$$

and 〈V〉 denotes the average membrane voltage across the population.

What remains in Equation 26 are equations for evaluating $\mu_{g_{s}}$ and $\sigma_{g_{s}}$. To derive the equation for evaluating $\mu_{g_{s}}=\int_{0}^{\infty }g_{s}\rho_{s}\left(g_{s},t\right)dg_{s}$, we multiply Equation 16 by *g_s_* and integrate *g_s_* from 0 to *∞*:

$$\frac{d}{dt}\mu_{g_{s}}=-\int_{0}^{\infty }g_{s}\frac{\partial }{\partial g_{s}}{\bar{J}}_{s}\left(g_{s},t\right)dg_{s}$$

$$=-g_{s}{\bar{J}}_{s}\left(g_{s},t\right){|}_{g_{s}=0}^{g_{s}=\infty }+\int_{0}^{\infty }\left[\frac{-g_{s}\rho_{s}\left(g_{s},t\right)}{\tau_{s}}+c_{s}\nu_{s}\left(t\right)\int_{g_{s}-\Gamma_{s}/\tau_{s}}^{g_{s}}\rho_{s}\left(g\prime ,t\right)dg\prime \right]dg_{s}$$

$$=\int_{0}^{\infty }\frac{-g_{s}\rho_{s}\left(g_{s},t\right)}{\tau_{s}}dg_{s}+c_{s}\nu_{s}\left(t\right)\int_{0}^{\infty }\left[\int_{g_{s}-\Gamma_{s}/\tau_{s}}^{g_{s}}\rho_{s}\left(g\prime ,t\right)dg\prime \right]dg_{s}\;(\text{BCs})$$

$$=-\frac{1}{\tau_{s}}\mu_{g_{s}}+c_{s}\nu_{s}\left(t\right)\int_{0}^{\infty }\left[\int_{g\prime }^{g\prime +\Gamma_{s}/\tau_{s}}dg_{s}\right]\rho_{s}\left(g\prime ,t\right)dg\prime \;(\text{Fubini})$$

$$=-\frac{1}{\tau_{s}}\mu_{g_{s}}+c_{s}\nu_{s}\left(t\right)\frac{\Gamma_{s}}{\tau_{s}}\int_{0}^{\infty }\rho_{s}\left(g\prime ,t\right)dg\prime$$

(31) $$=-\frac{1}{\tau_{s}}\mu_{g_{s}}+c_{s}\nu_{s}\left(t\right)\frac{\Gamma_{s}}{\tau_{s}}.$$

Similarly, one can derive the equation for evaluating $\mu_{g_{s}^{2}}=\int_{0}^{\infty }g_{s}^{2}\rho_{s}\left(g_{s},t\right)dg_{s}$, yielding

(32) $$\frac{d}{dt}\mu_{g_{s}^{2}}=-\frac{2}{\tau_{s}}\mu_{g_{s}^{2}}+c_{s}\nu_{s}\left(t\right)\left(\frac{2\Gamma_{s}}{\tau_{s}}\mu_{g_{s}}+\frac{\Gamma_{s}^{2}}{\tau_{s}^{2}}\right).$$

For $\sigma_{g_{s}}$, we use the identity, $\sigma_{g_{s}}=\sqrt{\mu_{g_{s}^{2}}-\mu_{g_{s}}^{2}}$.

In the csPDM, the population firing rate is directly estimated by the probability flux across *V_c_*:

(33) $$r\left(t\right)={\bar{J}}_{V}\left(V_{c},{\tilde{\mu }}_{g},{\tilde{\sigma }}_{g},t\right).$$

The average membrane voltage, 〈V(t)〉, is computed by using its definition:

(34) $$〈V\left(t\right)〉=\int_{V_{lb}}^{V_{c}}V\rho_{V}\left(V,t\right)dV.$$

The boundary conditions for solving Equation 26 are assigned as:

$${\bar{J}}_{V}\left(V_{r}^{+},{\tilde{\mu }}_{g},{\tilde{\sigma }}_{g},t\right)-{\bar{J}}_{V}\left(V_{r}^{-},{\tilde{\mu }}_{g},{\tilde{\sigma }}_{g},t\right)=r\left(t-\tau_{ref}\right),$$

$${\bar{J}}_{V}\left(V_{lb},{\tilde{\mu }}_{g},{\tilde{\sigma }}_{g},t\right)=0,$$

$$\rho_{V}\left(V_{c},t\right)=0.$$

They are similar to what used in the fdPDM except for the last boundary condition, which means that no neuron locates at *V_c_* because the neurons whose voltages reach *V_c_* are reset to *V_r_* immediately.

When the STP property is included, we replace Γ*_s_* and $\Gamma_{s}^{2}$ with

(35) $$\Gamma_{s}\to \Gamma_{s}\mu_{u_{s}}\mu_{x_{s}},$$

(36) $$\Gamma_{s}^{2}\to \Gamma_{s}^{2}\mu_{u_{s}}^{2}\mu_{x_{s}}^{2},$$

in Equation 31, Equation 32 for evaluating $\mu_{g_{s}}$ and $\sigma_{g_{s}}$, where $\mu_{u_{s}}$ and $\mu_{x_{s}}$ are mean values of *u_s_* and *x_s_* across the neuronal population, respectively. Following mean-field equations proposed by Barak and Tsodyks (2007), we use the same equations for tracking $\mu_{u_{s}}$ and $\mu_{x_{s}}$ (i.e., Eq. 1 in Barak and Tsodyks, 2007):

(37) $$\frac{d}{dt}\mu_{u_{s}}=\frac{1}{\tau_{s,f}}\left(U_{s}-\mu_{u_{s}}\right)+\nu_{s}\left(t\right)U_{s}\cdot \left(1-\mu_{u_{s}}\right),$$

(38) $$\frac{d}{dt}\mu_{x_{s}}=\frac{1}{\tau_{s,r}}\left(1-\mu_{x_{s}}\right)-\nu_{s}\left(t\right)\mu_{x_{s}}\mu_{u_{s}}.$$

To sum up, unlike in the fdPDM, the network dynamics in the csPDM is described by a system consisting of a one-dimensional quasi-Fokker–Planck equation for tracking the marginal density, $\rho_{V}\left(V,t\right)$, and 2*m* ordinary differential equations for tracking the first two statistical moments of all synapse-associated variables, $\left\{g_{s}\right\}$, or 4*m* equations for statistical moments of $\left\{g_{s},u_{s},x_{s}\right\}$ if STP is included. csPDM is expected to be more computationally efficient than fdPDM because solving one-dimensional quasi-Fokker–Planck equation undoubtedly takes less time than high-dimensional partial differential equations. Basically, they are solved numerically. In the next section, we present the numerical method used for solving the quasi-Fokker–Planck equation.

### Numerical method for solving quasi-Fokker–Planck equation

To solve Equation 26, we use local Galerkin method (LGM; Cockburn and Shu, 1998; Xu and Shu, 2010). This method belongs to the discontinuous Galerkin methods and focuses on the solutions of partial differential equations with high order derivatives. We select it as the proposed numerical method because it has superior ability to handle discontinuous solutions (Huang et al., 2015) and parallelizability in computations (Biswas et al., 1994). First, in the LGM, an auxiliary variable, q(V,t), is introduced to rewrite the Equation 26 as followings:

(39) $$\frac{\partial }{\partial t}\rho_{V}\left(V,t\right)=-\frac{\partial }{\partial V}\left[{ℋ}_{0}\left(V,{\tilde{\mu }}_{g}\right)\rho_{V}\left(V,t\right)-D\left(V,{\tilde{\sigma }}_{g}\right)q\left(V,t\right)\right],$$

(40) $$q\left(V,t\right)=\frac{\partial }{\partial V}\rho_{V}\left(V,t\right).$$

As a consequence, the Equation 26 is transformed to a conservative hyperbolic equation.

### Discretization of space domain and basic notations

For a given bounded *V*-domain $I=\left[V_{lb},V_{c}\right]$, we divide it into *M* meshes with an identical length as follows:

(41) $$V_{lb}=v_{\frac{1}{2}}<v_{\frac{3}{2}}<\ldots <v_{M+\frac{1}{2}}=V_{c},$$

so that *V_r_* and *E_s_* are exactly certain grid points. We denote the subspace $I_{k}=\left(v_{k-\frac{1}{2}},v_{k+\frac{1}{2}}\right)$ (k=1,2,…,M) and its length $L=\left(V_{c}-V_{lb}\right)/M$. Using first-order polynomials as shape functions, the approximated values of $\rho_{V}\left(V,t\right)$ and q(V,t) within *I_k_* are defined by:

(42) $${\tilde{\rho }}_{V}\left(v,t\right)\equiv \phi_{1}\left(v\right){\tilde{\rho }}_{k-\frac{1}{2}}^{+}\left(t\right)+\phi_{2}\left(v\right){\tilde{\rho }}_{k+\frac{1}{2}}^{-}\left(t\right),$$

(43) $$\tilde{q}\left(v,t\right)\equiv \phi_{1}\left(v\right){\tilde{q}}_{k-\frac{1}{2}}^{+}\left(t\right)+\phi_{2}\left(v\right){\tilde{q}}_{k+\frac{1}{2}}^{-}\left(t\right),$$

$$v\in I_{k},\;\phi_{1}\left(v\right)=\frac{v_{k+\frac{1}{2}}-v}{L},\;\phi_{2}\left(v\right)=\frac{v-v_{k-\frac{1}{2}}}{L},$$

in which *ϕ*_1_ and *ϕ*_2_ are shape functions. ${\tilde{\rho }}_{k+\frac{1}{2}}^{-}\left(t\right)$ and ${\tilde{\rho }}_{k+\frac{1}{2}}^{+}\left(t\right)$ refers to the value of ${\tilde{\rho }}_{V}$ at $v_{k+\frac{1}{2}}$ from the left mesh *I_k_* and from right mesh *I_k_*_+1_, respectively. Due to the discontinuity at the interface of adjacent meshes, ${\tilde{\rho }}_{k+\frac{1}{2}}^{-}\neq {\tilde{\rho }}_{k+\frac{1}{2}}^{+}$ is thus possible. ${\tilde{q}}_{k+\frac{1}{2}}^{-}\left(t\right)$ and ${\tilde{q}}_{k+\frac{1}{2}}^{+}\left(t\right)$ are defined in the same way.

### Element equations and numerical fluxes

Substituting $\rho_{V}\left(V,t\right)$ and q(V,t) with approximated values ${\tilde{\rho }}_{V}\left(V,t\right)$ and $\tilde{q}\left(V,t\right)$, respectively, multiplying shape functions and integrating with respect to *v* over the mesh *I_k_*, we then obtain element equations for cell *I_k_*, given by

(44) L[120012][ρ˜˙k−12+ρ˜˙k+12−]=1L[−∫Ikℋ0(v,μ˜g)ρ˜dv∫Ikℋ0(v,μ˜g)ρ˜dv]+1L[∫IkD(v,σ˜g)q˜dv−∫IkD(v,σ˜g)q˜dv]+[f^k−12−f^k+12]+[−D(vk−12,σ˜g)q^k−12D(vk+12,σ˜g)q^k+12],

(45) $$\left[\begin{array}{c} {\tilde{q}}_{k-\frac{1}{2}}^{+}\\ {\tilde{q}}_{k+\frac{1}{2}}^{-} \end{array}\right]=\frac{1}{L}\left[\begin{array}{cc} 1 & 1\\ -1 & -1 \end{array}\right]\left[\begin{array}{c} {\tilde{\rho }}_{k-\frac{1}{2}}^{+}\\ {\tilde{\rho }}_{k+\frac{1}{2}}^{-} \end{array}\right]+\frac{2}{L}\left[\begin{array}{c} -{\hat{\rho }}_{k-\frac{1}{2}}\\ {\hat{\rho }}_{k+\frac{1}{2}} \end{array}\right],$$

in which the notation “^” means numerical fluxes at the interfaces between cells. We set ${\hat{\rho }}_{k\pm \frac{1}{2}}={\tilde{\rho }}_{k\pm \frac{1}{2}}^{+}$ and ${\hat{q}}_{k\pm \frac{1}{2}}={\tilde{q}}_{k\pm \frac{1}{2}}^{-}$, and employ upwind fluxes for ${\hat{f}}_{k\pm \frac{1}{2}}$, yielding

(46) f^k±12={ℋ0(vk±12,μ˜g)ρ˜k±12−if ℋ0(vk±12,μ˜g)>0ℋ0(vk±12,μ˜g)ρ˜k±12+if ℋ0(vk±12,μ˜g)<0.

According to the boundary conditions, we set ${\hat{f}}_{\frac{1}{2}}={\hat{q}}_{\frac{1}{2}}={\hat{\rho }}_{N+\frac{1}{2}}=0$, corresponding to ${\bar{J}}_{V}(V_{lb},t)=0,\;\frac{\partial }{\partial V}\rho_{V}\left(V_{lb},t\right)=0$, $\rho_{V}\left(V_{c},t\right)=0$, respectively, and enforcedly assign ${\hat{f}}_{r-\frac{1}{2}}$ as ${\hat{f}}_{r-\frac{1}{2}}+r(t-\tau_{ref})$ if $v_{r-\frac{1}{2}}=V_{r}$. The firing rate *r*(*t*) is given by ${\hat{f}}_{N+\frac{1}{2}}-D\left(v_{N+\frac{1}{2}},{\tilde{\sigma }}_{g}\right){\hat{q}}_{N+\frac{1}{2}}$. We use the backward Euler method (Cheng and Shu, 2007) to solve element equations as well as Equations 31–38 to ensure numerical stability. Note that one needs to re-calculate element equations at each time step because ${ℋ}_{0}$ and *D* are functions of time-dependent parameters, $\mu_{g_{e}}$ and $\sigma_{g_{e}}$, respectively.

### Slope limiters

A slope limiter is employed to guarantee the positivity of the density function (Huang et al., 2015). After progressing one time step with the backward Euler method, the ${\tilde{\rho }}_{k\pm \frac{1}{2}}^{\mp }$ across all meshes go through a slope limiter ΛΠ, which is defined by

(47) $$\Lambda \Pi \left(\left[\begin{array}{c} {\tilde{\rho }}_{k-\frac{1}{2}}^{+}\\ {\tilde{\rho }}_{k+\frac{1}{2}}^{-} \end{array}\right]\right)=\left[\begin{array}{c} {\bar{\rho }}_{k}-\psi \left({\tilde{\rho }}_{k-\frac{1}{2}}^{+}-{\bar{\rho }}_{k},{\bar{\rho }}_{k}-{\bar{\rho }}_{k-1},{\bar{\rho }}_{k+1}-{\bar{\rho }}_{k}\right)\\ {\bar{\rho }}_{k}+\psi \left({\tilde{\rho }}_{k+\frac{1}{2}}^{-}-{\bar{\rho }}_{k},{\bar{\rho }}_{k}-{\bar{\rho }}_{k-1},{\bar{\rho }}_{k+1}-{\bar{\rho }}_{k}\right) \end{array}\right],$$

where ${\bar{\rho }}_{k}$ is set as $\frac{1}{2}\left({\tilde{\rho }}_{k-\frac{1}{2}}^{+}+{\tilde{\rho }}_{k+\frac{1}{2}}^{-}\right)$. The *ψ* is the “minmod function” defined as:

(48) $$\psi \left(a_{1},a_{2},a_{3}\right)=\{\begin{array}{ll} s\cdot \underset{i}{\min}\left|a_{i}\right| & if\,\;s=sign\left(a_{1}\right)=sign\left(a_{2}\right)=sign\left(a_{3}\right)\\ 0 & otherwise \end{array}.$$

Generally, Equations 10, 16 are also solved numerically. We adopt the so-called discontinuous Galerkin method to solve them. The details of this method are not stated here. Please refer to Huang’s paper (Huang et al., 2015) for details about the numerical method.

### Quantification of simulation errors

MCSs are considered as the ground-truth of all simulation examples in this study; thereby, the differences of the fdPDM, MMFM, and csPDM in simulation results with the MSC are used to explore their performances. The following two quantities are used to quantify the simulation errors of the csPDM on the estimations of marginal conductance and voltage density functions, given by

(49) $$\eta_{g}^{\text{csPDM}}=\frac{\int_{\mu_{g_{s}}-6\sigma_{g_{s}}}^{\mu_{g_{s}}+6\sigma_{g_{s}}}\left|\rho_{s}^{\text{MCS}}\left(g_{s}\right)-N\left(g_{s}|\mu_{g_{s}},\sigma_{g_{s}}\right)\right|dg_{s}}{\int_{\mu_{g_{s}}-6\sigma_{g_{s}}}^{\mu_{g_{s}}+6\sigma_{g_{s}}}\rho_{s}^{\text{MCS}}\left(g_{s}\right)dg_{s}},$$

(50) $$\eta_{V}^{\text{csPDM}}=\frac{\int_{V_{lb}}^{V_{c}}\left|\rho_{V}^{\text{MCS}}\left(V\right)-\rho_{V}^{\text{csPDM}}\left(V\right)\right|dV}{\int_{V_{lb}}^{V_{c}}\rho_{V}^{\text{MCS}}\left(V\right)dV},$$

to check the validity of diffusion approximation on synaptic dynamics. $\eta_{g}^{\text{csPDM}}$ describes the average error ratio on the marginal conductance density function, which is estimated only over the interval $\left(\mu_{g_{s}}-6\sigma_{g_{s}},\mu_{g_{s}}+6\sigma_{g_{s}}\right)$. It is noted that the marginal conductance density function in the csPDM is characterized by a Gaussian distribution, here denoted by $N\left(g_{s}|\mu_{g_{s}},\sigma_{g_{s}}\right)$, whose mean and standard deviation are $\mu_{g_{s}}$ and $\sigma_{g_{s}}$. $\eta_{V}^{\text{csPDM}}$ is the average error ratio on the marginal voltage density function.

The third quantity is

(51) $$\eta_{r}^{*}=\frac{\int_{t_{1}}^{t_{2}}\left|r^{\text{MCS}}\left(t\right)-r^{*}\left(t\right)\right|dt}{\int_{t_{1}}^{t_{2}}r^{\text{MCS}}\left(t\right)dt},$$

where *∈{fdPDM,MMFM,csPDM}. It is used for comparing performances of the fdPDM, MMFM, and csPDM. $\eta_{r}^{*}$ means the average error ratio on the population firing rate obtained from fdPDM, MMFM, or csPDM. $r^{\text{MCS}}\left(t\right)$ is calculated from Equation 8. $r^{*}\left(t\right)$ is calculated from Equation 13 for fdPDM, Equation 18 for MMFM, and Equation 33 for csPDM.

## Results

We will present four simulation examples: (1) steady-state analyses, (2) dynamic population responses to excitatory input only, (3) dynamic population responses to multiple inputs, and (4) STP population responses. Using the former two examples, we aim to compare the performances of fdPDM, MMFM, and csPDM and focus on the accuracy and efficiency of csPDM. We show the applicability of csPDM to more complicated neuronal activities by the latter two examples. Thus, in both the examples, only the simulation results of MCS and cdPDM are presented. The parameters required for simulations are listed inTable 2. The values of neuronal properties are assigned according to previous studies (Fourcaud-Trocmé et al., 2003; Destexhe, 2009; Hertäg et al., 2014), and all of them are within in the physiologic ranges. The parameters relevant to synaptic dynamics and STP are regarded as free parameters which are freely tuned to let *τ_s_* correspond to the time scales of common transmitter receptors or to make synaptic interactions exhibit short-term facilitation/depression if necessary. The input rate, *ν_s_*, shown in this section is normalized by the minimum constant input rate that drives EIF neurons to produce spikes under fluctuation-free conditions (i.e., mean-field models), except for the third and fourth simulation examples. Such a minimum input rate is $g_{l}\left(V_{T}-E_{l}-\kappa \right)/\left[\left(E_{s}-V_{T}\right)\Gamma_{s}c_{s}\right]$. Next, we start to show simulation results by first checking the validity condition of diffusion approximation of synaptic dynamics.

**Table 1:** List of notation and symbols

Name	Symbol	SI unit
Average membrane voltage across population	〈V〉	V
Average error ratio of csPDM on marginal conductance density function	$\eta_{g}^{\text{csPDM}}$	Dimensionless
Average error ratio of csPDM on marginal voltage density function	$\eta_{V}^{\text{csPDM}}$	Dimensionless
Average error ratio on population firing rate	$\eta_{r}^{*}$	Dimensionless
Autocorrelation function of synaptic conductance	$C_{s}$	
Conductance probability flux	$J_{g_{s}}$	
Conductance probability flux vector	${\overset{˜}{J}}_{g}$	
Conductance state vector	$\overset{˜}{g}$	
Cutting voltage	*V_c_*	V
Facilitation time constant	*τ_s_*_,_*_f_*	s
Fluctuation velocity on neuronal Langevin equation	${ℋ}_{1}\left(V,{\overset{˜}{\sigma }}_{g},\overset{˜}{\overset{¯}{g}}\right)$	
Initial utilization parameter	*U_s_*	Dimensionless
Instant voltage jump	$\Delta V_{s}$	V
Leak conductance	*g_l_*	S
Leak reversal potential	*E_l_*	V
Lower bound on voltage direction	*V_lb_*	V
Marginal conductance density function	$\rho_{s}\left(g_{s},t\right)$	
Marginal conductance probability flux	${\bar{J}}_{s}$	
Marginal voltage density function	$\rho_{V}\left(V,t\right)$	
Marginal voltage probability flux	${\bar{J}}_{V}$	
Mean-field velocity on neuronal Langevin equation	${ℋ}_{0}\left(V,{\tilde{\mu }}_{g}\right)$	
Mean of conductance state vector	${\tilde{\mu }}_{g}$	
Mean of running utilization parameter	$\mu_{u_{s}}$	Dimensionless
Mean of running fraction of available neurotransmitters	$\mu_{x_{s}}$	Dimensionless
Mean of synaptic conductance	$\mu_{g_{s}}$	S
Mean rate of external Poisson input	*ν_s_*	Hz
Membrane voltage	*V*	V
Membrane capacitance	*C*	F
Neuronal velocity component along voltage direction	$F_{V}\left(V,\tilde{g}\right)$	
Number of synaptic connections	*c_s_*	
Population density function	$\rho \left(V,\tilde{g},t\right)$	
Population firing rate	r(t)	Hz
Recovery time constant	$\tau_{s,r}$	s
Refractory period	*τ_ref_*	s
Resetting voltage	*V_r_*	V
Running fraction of available neurotransmitters	*x_s_*	Dimensionless
Running utilization parameter	*u_s_*	Dimensionless
Sharpness factor	*κ*	V
Standard deviation of synaptic conductance	$\sigma_{g_{s}}$	S
Standard deviation of synaptic conductance state vector	${\tilde{\sigma }}_{g}$	
Steady firing rate for a conductance vector	$\bar{r}\left(\tilde{g}\right)$	Hz
Stochastic diffusion tensor	$D\left(V,{\tilde{\sigma }}_{g}\right)$	
Stochastic Ornstein–Uhlenbeck conductance	${\bar{g}}_{s}$	
Stochastic Ornstein–Uhlenbeck conductance state vector	$\tilde{\bar{g}}$	
Synaptic current	$I_{\text{syn}}\left(V,t\right)$	A
Synaptic conductance	*g_s_*	S
Synaptic reversal potential	*E_s_*	V
Synaptic time constant	*τ_s_*	s
Threshold voltage	*V_T_*	V
Total variation of synaptic conductance	Γ*_s_*	S
Voltage probability flux	*J_V _*	
White Gaussian noise with unit variance	ξ(t)	

**Table 2:** List of values of model parameters

Neuronal properties					
*C*	1 *μ*F	*g_L_*	0.05 mS	*E_L_*	–65 mV
*V_T_*	–50 mV	*κ*	2 mV	*V_c_*	–40 mV
*V_r_*	–65 mV	*τ_ref_*	3 ms		
AMPA-mediated synaptic dynamics					
$\Delta V_{\text{s}}$	1 mV	*τ_s_*	5 ms	*E_s_*	0 mV
GABA_A_-mediated synaptic dynamics					
$\Delta V_{\text{s}}$	0.25 mV	*τ_s_*	10 ms	*E_s_*	–80 mV
GABA_B_-mediated synaptic dynamics					
$\Delta V_{\text{s}}$	0.25 mV	*τ_s_*	100 ms	*E_s_*	–100 mV
Short-term facilitation					
$\Delta V_{\text{s}}$	2 mV	*U_s_*	0.05	$\tau_{s,f}$	700 ms
$\tau_{s,r}$	100 ms				
Short-term depression					
$\Delta V_{\text{s}}$	2 mV	*U_s_*	0.2	$\tau_{s,f}$	50 ms
$\tau_{s,r}$	300 ms				

### Validation of diffusion approximation of synaptic dynamics

**Figure 1.**
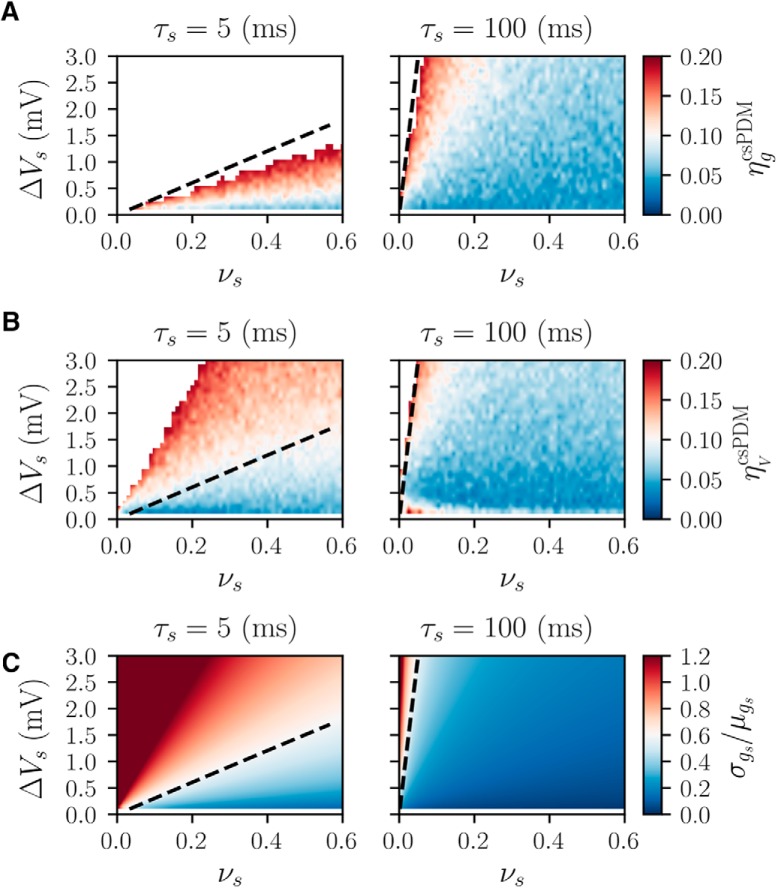
Steady-state values of $\eta_{g}^{\text{csPDM}}$, $\eta_{V}^{\text{csPDM}}$ and $\sigma_{g_{s}}/\mu_{g_{s}}$ of a single population with respect to different *ν_s_* and $\Delta V_{s}$ ***A***, $\eta_{g}^{\text{csPDM}}$ ***B***, $\eta_{V}^{\text{csPDM}}$ ***C***, $\sigma_{g_{s}}/\mu_{g_{s}}$. Black dashed lines indicate the sets of (*ν_s_*, Δ*V_s_*) where $\sigma_{g_{s}}/\mu_{g_{s}}=0.6$. Blank areas in ***A***, ***B*** indicate the error ratio of >0.2.

**Figure 2.**
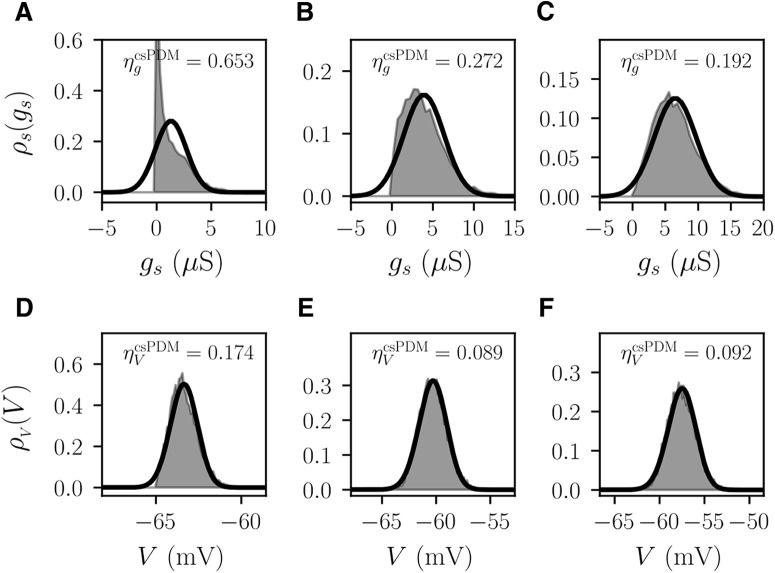
Examples of steady-state marginal conductance density functions $\rho_{s}\left(g_{s}\right)$ and marginal voltage density functions $\rho_{V}\left(V\right)$. ***A***, ***D***, $\rho_{s}\left(g_{s}\right)$ and $\rho_{V}\left(V\right)$ under $\nu_{s}=0.1$ and $\Delta V_{s}=1$ mV. ***B***, ***E***, $\rho_{s}\left(g_{s}\right)$ and $\rho_{V}\left(V\right)$ under $\nu_{s}=0.3$ and $\Delta V_{s}=1$ mV. ***C***, ***F***, $\rho_{s}\left(g_{s}\right)$ and $\rho_{V}\left(V\right)$ under $\nu_{s}=0.5$ and $\Delta V_{s}=1$ mV. As shown, the estimations of $\rho_{s}\left(g_{s}\right)$ and $\rho_{V}\left(V\right)$ by csPDM are similar with those by MCS if the input rate *ν_s_* is increased. *τ_s_* is set as 5 ms in all examples. Shaded area: MCS. Black thick line: csPDM.

For the correct use of the csPDM, to explore the validity condition of diffusion approximation of synaptic dynamics is necessary. To do so, we check steady-state values of $\eta_{g}^{\text{csPDM}}$ and $\eta_{V}^{\text{csPDM}}$ of a single neuronal population receiving only constant excitatory inputs with respect to different *ν_s_* and $\Delta V_{s}$. The synaptic time constant *τ_s_* is set as 5 or 100 ms to match the time scale of AMPA-receptors or NMDA-receptors (Dayan and Abbott, 2001), and *E_s_* is set as 0 mV for both cases. Basically, small $\eta_{g}^{\text{csPDM}}$ reflects the validity of diffusion approximation. As shown in Figure 1*A*, larger *ν_s_* or smaller $\Delta V_{s}$ results in smaller $\eta_{g}^{\text{csPDM}}$ for both *τ_s_* = 5 and *τ_s_* = 100 ms, in agreement with the fact that the diffusion approximation is valid when the input rate is large and the jump size is small. As seen, increasing *τ_s_* produces small $\eta_{g}^{\text{csPDM}}$ because of the decreased variation of *g_s_* ($\sigma_{g_{s}}\propto 1/\sqrt{\tau_{s}}$ at steady states). Three distributions of *g_s_* under different sets of (*ν_s_*, $\Delta V_{s}$) are shown in Figure 2*A–C*. As shown, the distribution of *g_s_*, $\rho_{s}\left(g_{s}\right)$, approaches a Gaussian distribution when $\eta_{g}^{\text{csPDM}}$ is <0.2 (Fig. 2*C*). That is, the diffusion approximation of synaptic dynamics is valid if $\eta_{g}^{\text{csPDM}}<0.2$. Figure 1*B* shows $\eta_{V}^{\text{csPDM}}$. The amplitude of $\eta_{V}^{\text{csPDM}}$ is proportional to that of $\eta_{g}^{\text{csPDM}}$, meaning that the error of csPDM in $\rho_{V}\left(V\right)$ in large part comes from the failure of the diffusion approximation. But, surprisingly, the value of $\eta_{V}^{\text{csPDM}}$ is much lower than that of $\eta_{g}^{\text{csPDM}}$ under the same set of (*ν_s_*, Δ*V_s_*). As seen in Figure 2*A*,*D*, under the case where $\nu_{s}=0.1,\;\Delta V_{s}=1$ mV, and *τ_s_* = 5 ms, $\eta_{V}^{\text{csPDM}}$ is only 0.174 whereas $\eta_{g}^{\text{csPDM}}$ is as high as 0.653. However, the reason why $\eta_{V}^{\text{csPDM}}$ is smaller than $\eta_{g}^{\text{csPDM}}$ is unclear. Through the observation of $\rho_{V}\left(V\right)$ (Fig. 2*D–F*), it is found that csPDM gives an adequately accurate estimation of $\rho_{V}\left(V\right)$ comparable to MCS when $\eta_{V}^{\text{csPDM}}$ is <0.2. To offer a quantitative and universal validity condition for diffusion approximation of synaptic dynamics, we also check the value of $\sigma_{g_{s}}/\mu_{g_{s}}$ (i.e., the coefficient of variation of *g_s_*; Fig. 1*C*). In fact, it has been argued that the diffusion approximation is valid only when $\sigma_{g_{s}}/\mu_{g_{s}}<<1$, meaning that the conductance mean $\mu_{g_{s}}$ should be much larger than the standard deviation $\sigma_{g_{s}}$ (Richardson and Gerstner, 2005; Cai et al., 2012). It is however an impractical condition. Based on our observations of $\eta_{g}^{\text{csPDM}}$ and $\eta_{V}^{\text{csPDM}}$, it can be conclusively said that the validity condition of diffusion approximation for the application of csPDM is $\sigma_{g_{s}}/\mu_{g_{s}}\leq 0.6$, under which $\eta_{g}^{\text{csPDM}}$ and $\eta_{V}^{\text{csPDM}}$ are approximately <0.2. As a result, Δ*V_s_* should be <1.5 mV for the case of *τ_s_* = 5 ms such that this criterion can be satisfied in the range of $\nu_{s}\geq 0.6$. In the following simulations, we choose Δ*V_s_* as 1 mV for the excitatory inputs.

### Steady-state analyses

**Figure 3.**
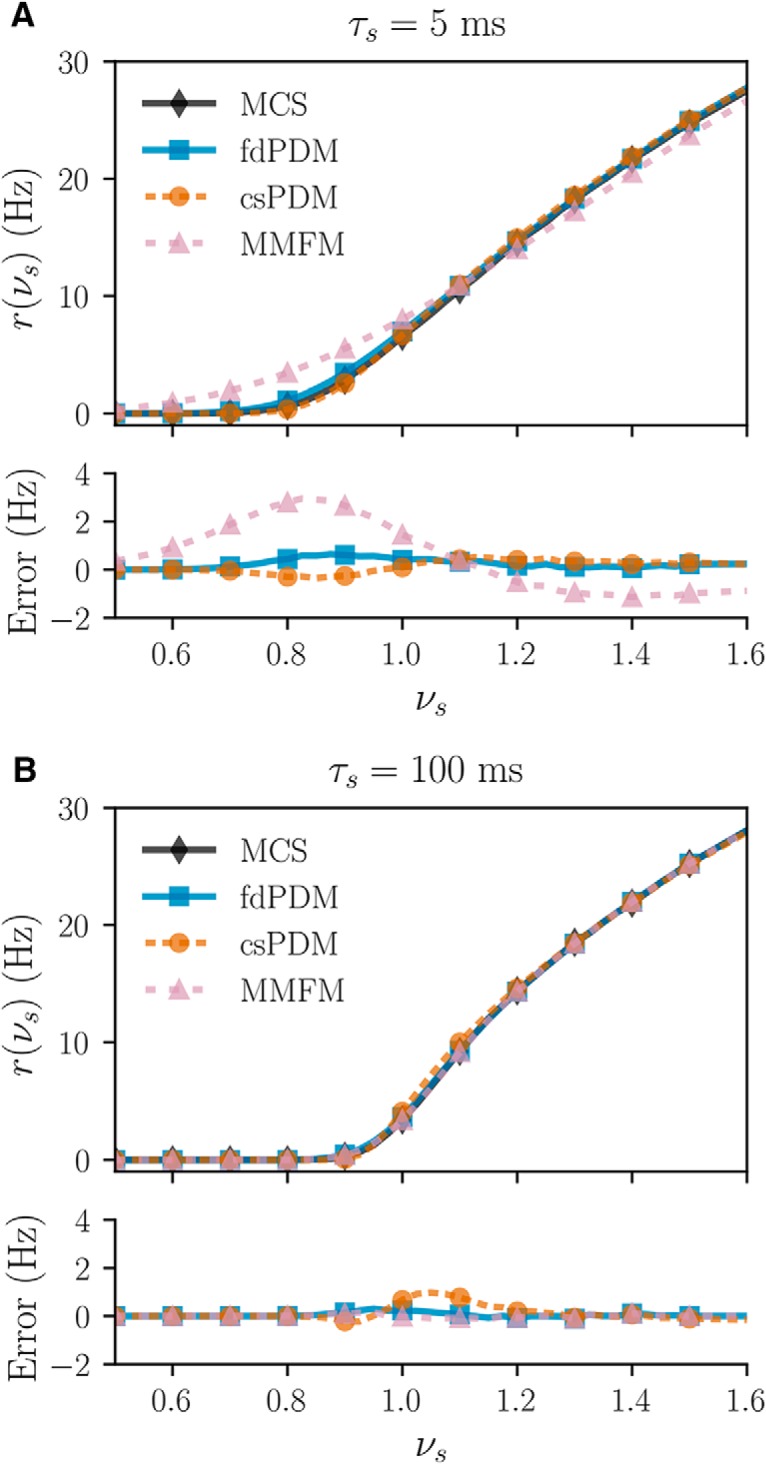
Input-output curve comparison. The top panels in ***A***, ***B*** show the input-output curves computed in four ways: MCS (black solid line with diamond markers), fdPDM (blue solid line with square markers), csPDM (orange dashed line with circle markers), and MMFM (pink dashed line with triangle markers). Synaptic time constant (*τ_s_*) is 5 ms in ***A*** and is 100 ms in ***B***. $r\left(\nu_{s}\right)$ is the steady-state population firing rate as a function of a fixed input rate *ν_s_*. The bottom panels in ***A***, ***B*** show the errors of fdPDM, csPDM, and MMFM in $r\left(\nu_{s}\right)$ from MCS. Δ*V_s_* = 1 and *E_s_* = 0 mV in this example.

The performances of fdPDM, MMFM, and csPDM are first examined via steady-state analyses of population firing rates in response to fixed excitatory inputs. Here, we also consider two cases, *τ_s_* = 5 and *τ_s_* = 100 ms, and set Δ*V_s_* = 1 mV and *E_s_* = 0 mV. Figure 3 displays input-output curves computed by MSC, fdPDM, csPDM, and MMFM. Results show that csPDM gives an estimation of input-output curves that are close to those from MSC and fdPDM with errors <1 Hz for both *τ_s_* = 5 and *τ_s_* = 100 ms. These results indicate that csPDM can accurately estimate steady-state output population firing rates although it just tracks the marginal voltage density function. In contrast, MMFM gives accurate estimations of steady-state output firing rates only under the condition of *τ_e_* = 100 ms. It overestimates the actual firing rate in the fluctuation-driven regime (i.e., *ν_s_* < 1) and underestimates the actual firing rate in the mean-driven regime (i.e., *ν_s_* > 1) in the case of *τ_s_* = 5 ms (Fig. 3*A*, bottom panel, pink dashed line with triangle markers). The overestimation or underestimation of the MMFM in the case of *τ_e_* = 5 ms was also observed by Ly (2013). However, the demonstration that such overestimation or underestimation disappears when the synaptic time constant increases has not been reported.

### Dynamic population responses to excitatory input only

**Figure 4.**
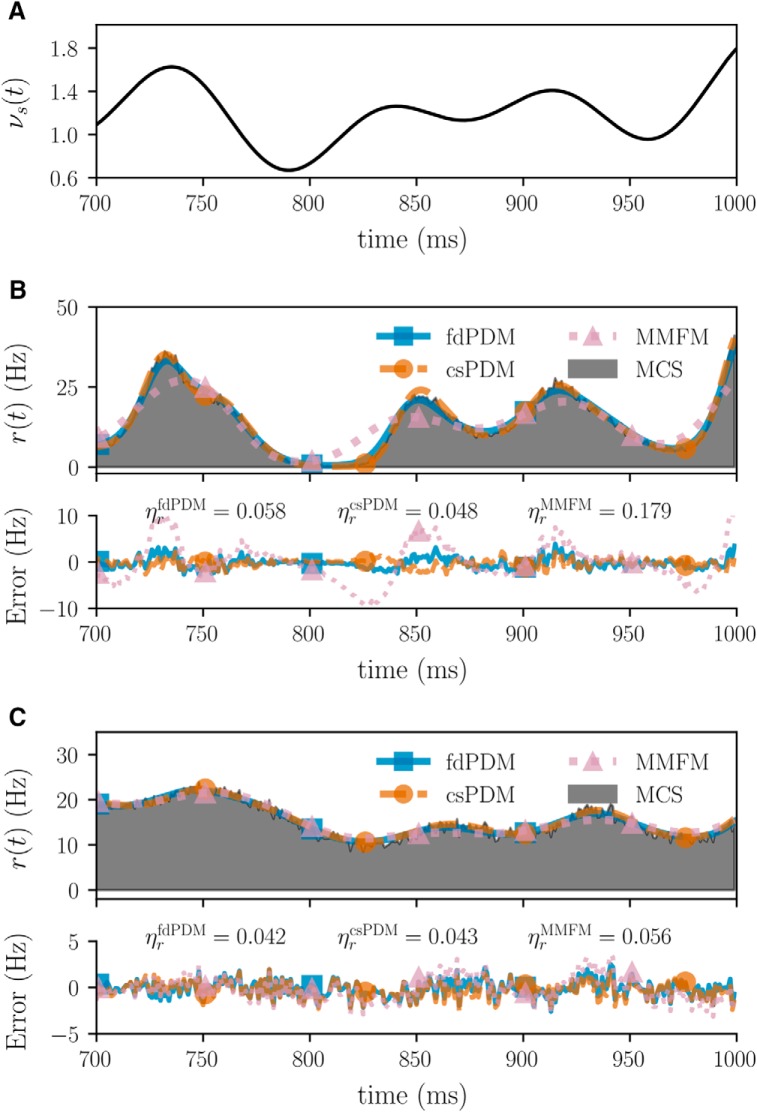
Dynamic population responses to a time-varying excitatory input. ***A***, Time-varying input rate *ν_s_*. ***B***, top panel, Population responses *r*(*t*) in response to *ν_s_* computed by MCS (shaded area), fdPDM (blue solid line with square markers), csPDM (orange dashed line with circle markers), and MMFM (pink dotted line with traiangle markers) under *τ_s_* = 5 ms. Bottom panel, Corresponding errors of fdPDM, csPDM, and MMFM in *r*(*t*) from MCS. ***C***, Similar to ***B*** except for *τ_s_* = 100 ms. Average error ratios $\eta_{r}^{*}$ were computed over the interval from 600 and 1000 ms. $\eta_{r}^{\text{fdPDM}},\;\eta_{r}^{\text{csPDM}}$, and $\eta_{r}^{\text{MMFM}}$ are 0.058, 0.048, and 0.179 under *τ_s_* = 5 ms, respectively. They are 0.042, 0.043, and 0.056 for *τ_s_* = 100 ms. Δ*V_s_* = 1 and *E_s_* = 0 mV in this example.

In addition to the steady-state analyses described in the above, the comparison between csPDM and MMFM is also made by investigating their ability to capture dynamic population firing rates in response to time-varying inputs. This is the most essential test to reveal whether they are adequate as dimension-reduction methods. Similar to the previous cases, excitatory inputs with *τ_s_* = 5 and *τ_s_* = 100 ms are considered here. The time-varying input rate is shown in Figure 4*A*, which varies in time and takes values that guarantee the validity of diffusion approximation, i.e., $\nu_{s}\geq 0.6$. Figure 4*B* shows the output population firing rates *r*(*t*) computed by MCS, fdPDM, csPDM, and MMFM and the corresponding errors in *r*(*t*) for *τ_s_* = 5 ms. Figure 4*C* is similar to Figure 4*B* except *τ_s_* = 100 ms. As shown in these two panels, whatever synaptic time constant is, csPDM accurately captures all the qualitative features of the output firing rates, leading to small average error ratios, which are 0.048 and 0.043 for *τ_s_* = 5 and *τ_s_* = 100 ms. It also gives comparable simulation results to those of fdPDM (similar average error ratios between fdPDM and csPDM). However, like in the steady-state analyses, MMFM gives accurate output firing rates only when *τ_s_* = 100 ms. It overestimates the low output firing rates (at about *t* = 820 ms) and underestimates the high output firing rates (at about *t* = 730 or *t* = 850 ms) when *τ_s_* = 5 ms, leading to a large error ratio $\eta_{r}^{\text{MMFM}}$ of 0.179.

**Figure 5.**
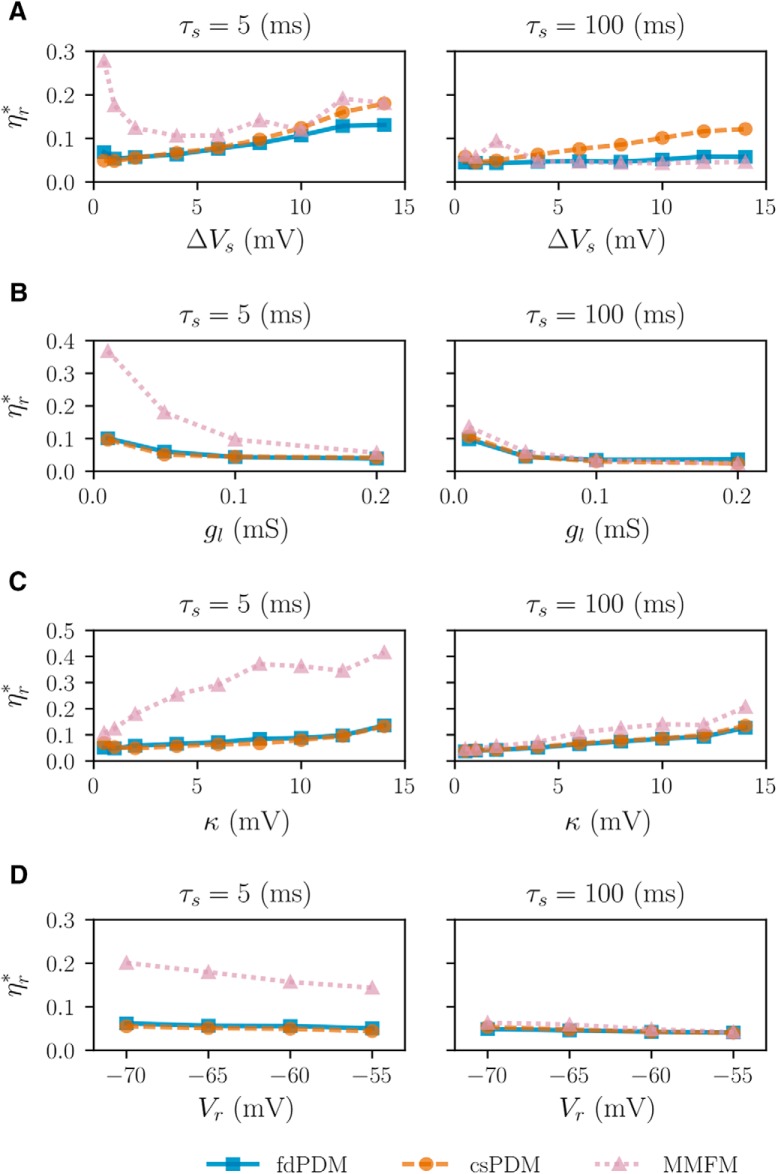
Average error ratios of fdPDM, csPDM, and MMFM in population responses under different parameter sets. ***A***, Subject to varying Δ*V_s_*. ***B***, Subject to varying *g_l_*. ***C***, Subject to varying *κ*. ***D***, Subject to varying *V_r_*. Blue solid line with square markers: fdPDM. Orange dashed line with circle markers: csPDM. Pink dotted line with traiangle markers: MMFM. $\eta_{r}^{*}$ means average error ratios corresponding to fdPDM, csPDM, and MMFM.

**Figure 6.**
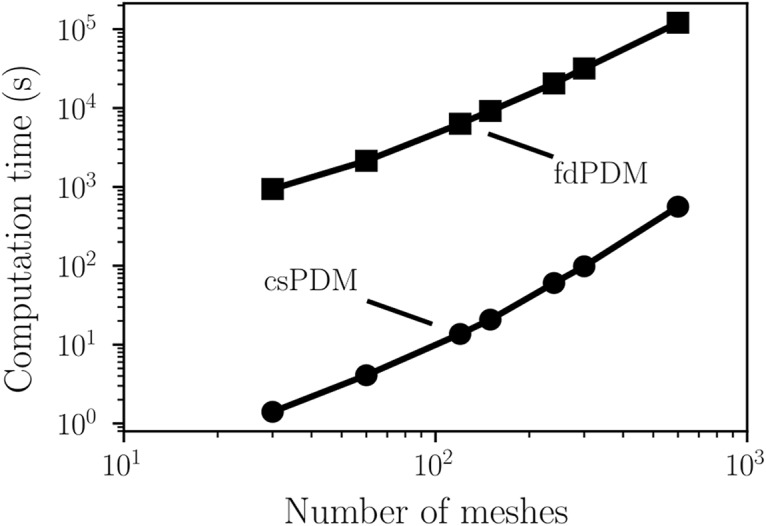
Comparison of computational time between fdPDM and csPDM. As shown, the computational speed of csPDM is almost 1000 times faster than fdPDM.

To show how robust the csPDM is, we explore whether the accuracy of csPDM depends on the values of model parameters. Four parameters, Δ*V_s_*, *g_l_*, *κ* and *V_r_*, are chosen for this test. The same input rate as shown in the Figure 4*A* is considered as the input. As shown in Figure 5*A*, augmenting the jump size Δ*V_s_* increases $\eta_{r}^{\text{csPDM}}$, meaning that the accuracy of csPDM is decreased. This is due to the fact that diffusion approximation becomes invalid when Δ*V_s_* is too large (as shown in Fig. 1). As expected, $\eta_{r}^{\text{fdPDM}}$ is less sensitive to the change of Δ*V_s_* than $\eta_{r}^{\text{csPDM}}$ because Δ*V_s_* is arbitrary in the fdPDM. Surprisingly, although MMFM does not have limitations on Δ*V_s_*, in the case of *τ_s_* = 5 ms, $\eta_{r}^{\text{MMFM}}$ severely fluctuates over a range of 0.2 when changing Δ*V_s_*. Figure 5*B–D*unravel that changing *g_l_*, *κ* and *V_r_* almost does not affect the accuracy of csPDM ($\eta_{r}^{\text{csPDM}}$ is below 0.1 in most parameter sets), implying that csPDM is a robust method. Importantly, simulation results from csPDM are comparable to those from fdPDM. MMFM can give comparable results to csPDM only when *τ_s_* = 100 ms. However, in the case of *τ_s_* = 5 ms, reducing *g_l_* enlarges the error ratio $\eta_{r}^{\text{MMFM}}$ with an increasing magnitude of 0.3 (from 0.07 to 0.38). So does increasing *κ* (from 0.1 to 0.4). In other words, MMFM is not a robust method for EIF models. Figure 6 shows the computational time spent by fdPDM and csPDM subject to different numbers of meshes employed in numerical methods for a simulation of 1 s. The numerical simulation used in this test is the same as shown in Figure 4. Remarkably, the computational efficiency of csPDM is ∼1000 times better than fdPDM. Low computational efficiency of fdPDM certainly comes from the existence of the *g_s_*-dimension in the master equation because, except for the requirement of more grid meshes along this dimension (We set 120 meshes along this dimension), its existence leads to the necessity of extremely small time steps for satisfying the Courant–Friedrichs–Lewy condition to ensure numerical stability of discontinuous Galerkin methods (Huang et al., 2015). In this case, the time step is 0.02 ms for fdPDM but 0.2 ms for csPDM.

In the next two examples, we only use csPDM to estimate the population dynamics and compare the results with those of MCS to evaluate its performance because, via the examples above, it has been demonstrated that, compared to fdPDM and MMFM, only csPDM can provide simulation results efficiently and accurately.

### Dynamic population responses with excitatory and inhibitory inputs

To highlight the outstanding ability of csPDM to achieve dimension reduction, here, we consider a real case where a population of cortical pyramidal neurons receives excitatory inputs from the neighboring pyramidal neuronal population mediated by AMPA receptors and, meanwhile, inhibitory inputs from the interneuron population mediated by two types of receptors, GABA_A_ and GABA_B_ (Suffczynski et al., 2004; Cona et al., 2014). To mimic this situation, we consider a single uncoupled population of EIF neurons, representing pyramidal neurons, which receive external excitatory inputs mediated by AMPA-type receptors, which have *τ_s_* = 5 ms and Δ*V_s_* = 1 mV, and inhibitory inputs mediated by GABA_A_-type receptors, which have *τ_s_* = 10 ms and Δ*V_s_* = 0.25 mV, as well as GABA_B_-type receptors, which have *τ_s_* = 100 ms and Δ*V_s_* = 0.25 mV. Each neuron is assumed to have 200 excitatory and 200 inhibitory synaptic connections.

**Figure 7.**
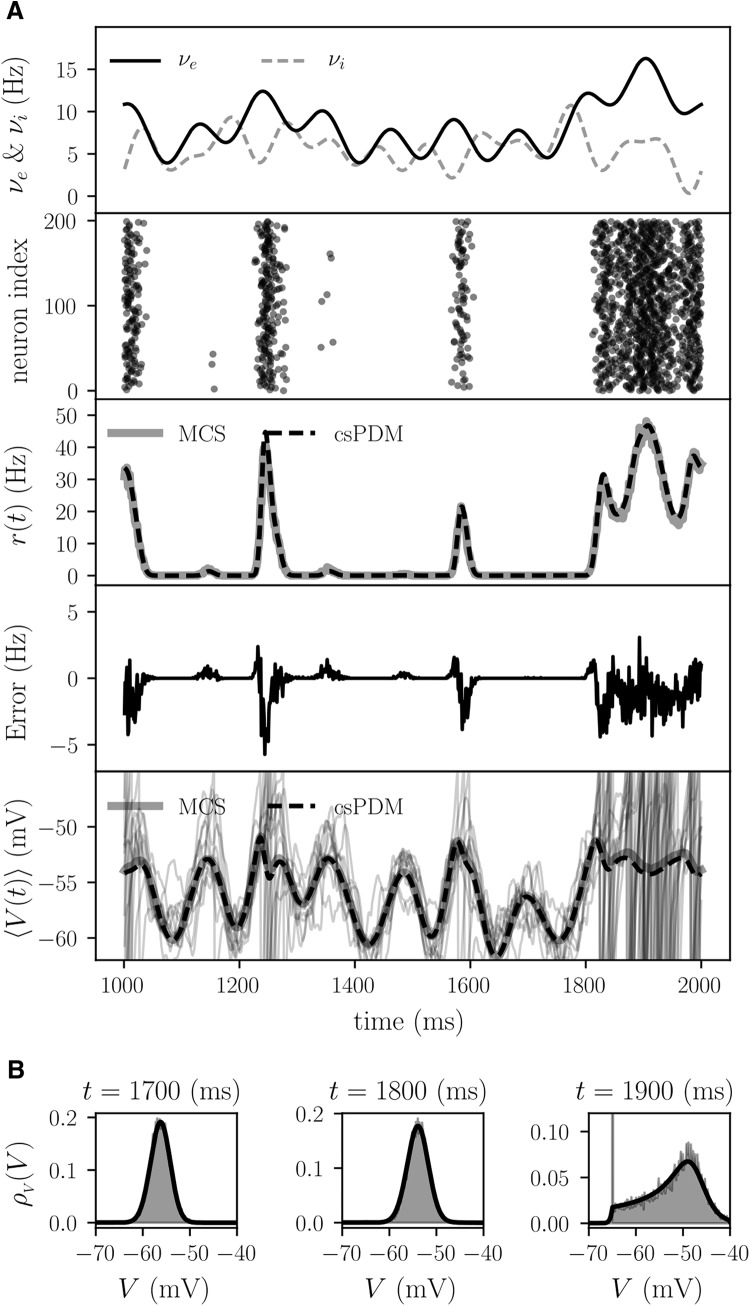
MCS and csPDM for a single population receiving excitatory and inhibitory inputs gated by three types of neurotransmitter receptors. ***A***, The panels from top to bottom show (1) time-varying excitatory and inhibitory input rates (*ν_e_* denotes the excitatory input rate, and *ν_i_* denotes the inhibitory one); (2) raster plot of output spikes from 200 neurons in MCS; (3) the population firing rates *r*(*t*) computed by MCS (gray solid line) and csPDM (black dashed line); (4) the error in *r*(*t*) between MCS and csPMD; and (5) average membrane voltages across the population *r*(*t*) computed by MCS (gray thick solid line) and csPDM (black thick dashed line). Gray thin solid lines: voltage traces from 10 neurons in MSC. ***B***, Three snapshots of the marginal density function $\rho_{V}\left(V,t\right)$ at t = 1700 , 1800, and 1900 ms from left to right. Shaded area: MCS. Black thick line: csPDM.

Figure 7*A*, top panel, shows the time-varying input rates on excitatory and inhibitory synaptic connections. It should be noted that the inputs gated by GABA_A_ and GABA_B_ receptors share the common input rate in statistical sense. The Figure 7*A*, second panel from top, is the raster plot of output spikes from 200 neurons in MCS. With respect to the output population firing rate, as seen in Figure 7*A*, third panel, csPDM is able to capture the salient features of the population firing rate. The average error ratio $\eta_{r}^{\text{csPDM}}$ is ∼ 0.067 (below 0.1). It is ∼ 4 Hz of error in the high output firing rate (such as at *t* = 1900 ms; Fig. 7*A*, forth panel) on average. csPDM also can accurately estimate the average membrane voltage across the population (Fig. 7*A*, fifth panel). Figure 7*B* shows the snapshots of the marginal density function $\rho_{V}\left(V,t\right)$ at *t* = 1700, 1800, and 1900 ms. As seen, the density functions computed by MCS and csPDM match well in all three snapshots. The corresponding values of $\eta_{V}^{\text{csPDM}}$ are 0.065, 0.07, and 0.122, respectively. These results indicate that $\rho_{V}\left(V,t\right)$ can be correctly captured by csPDM.

### STP population responses

**Figure 8.**
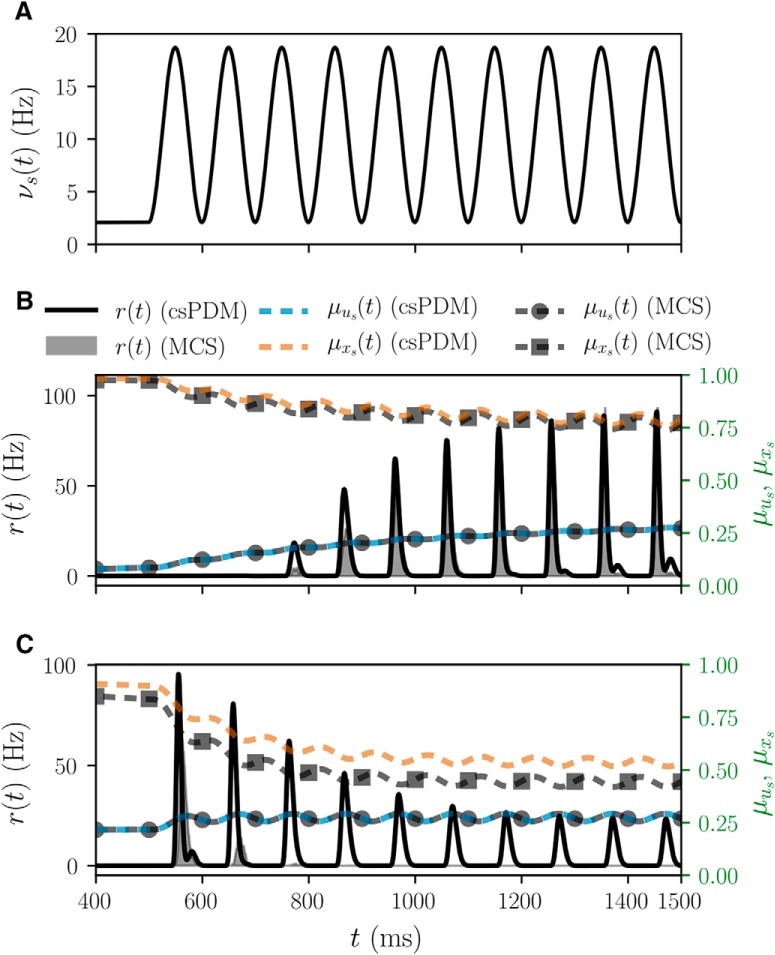
STP population responses. ***A***, Time-varying excitatory input rate. ***B***, Population responses under short-term facilitation. The population response is facilitated during the sinusoidal input, resulting from the sharp increase in the running utilization parameter, $\mu_{u_{s}}$ As shown, the csPDM can capture the STP property of the population responses not only qualitatively but also quantitatively. ***C***, Population responses under short-term depression. The population response is depressed, resulting from the sharp decrease in the running fraction of available neurotransmitters, $\mu_{x_{s}}$. In this case, csPDM only captures the STP property of the population responses qualitatively. The larger error in population responses is due to the overestimation of $\mu_{x_{s}}$

In this part, we test the effects of the synaptic STP on population responses to see if csPDM can capture these effects. We consider a single population of EIF neurons receiving only excitatory inputs through plastic synapses with short-term facilitation or depression. For the facilitative synapses, we adopt *τ_s_* = 5 ms, $\Delta V_{s}=2$ mv *E_s_* = 0 mV, *τ_s_*_,_*_f_* = 700 ms, *τ_s_*_,_*_r_* = 100, and *U_s_* = 0.05. They are the same except *τ_s_*_,_*_f_* = 50 ms, *τ_s_*_,_*_r_* = 300, and *U_s_* = 0.2 for the depressive synapses. Figure 8*A* shows the time-varying input rate, which consists of a fixed baseline of 500 ms and, afterward, a sinusoidal fluctuation of 1 s. The population responses and ensemble averages of *u_s_* and *x_s_* obtained from MSC and csPDM for the case of short-term facilitation are plotted in Figure 8*B*. As seen, the population response gradually increases in the latter cycles in the sinusoidal fluctuation (800–1500 ms). The facilitative response actually stems from the sharp increase in $\mu_{u_{s}}$ from 0.05 to ∼0.29. Meanwhile, the value of $\mu_{x_{s}}$ remains at a high level ($\mu_{x_{s}}\approx 0.76$, eventually). Figure 8*C* is similar to Figure 8*B* except that depressive synapses are used. As shown, the population response is depressed along the sinusoidal input, resulting from the large decrease in $\mu_{x_{s}}$ roughly by 0.4, during which $\mu_{u_{s}}$ remains almost fixed. Note that, different to the above example, where csPDM can give quantitatively accurate estimates of population responses, in this case, csPDM just captures STP property of population responses qualitatively. As seen in Figure 8*C*, the output firing rate obtained from csPDM drops but does not vanishes, while it vanishes in MCS. This difference is caused by the mismatch of $\mu_{x_{s}}$ between MSC and csPDM. In summary, the population response can inherit the STP property from synapses, and csPDM captures such property qualitatively.

## Discussion

In this study, we present a principled and straightforward dimension-reduction method for PDM to handle realistic synaptic dynamics and compare it with another dimension-reduction method, called MMFM proposed by Ly (2013). We name the newly proposed reduction method csPDM. The csPDM does not assume specific limits on synaptic time constants so that we can consider synaptic dynamics mediated by all kinds of common receptors, including AMPA, GABA_A_, GABA_B_, and even NMDA. Through our examples, it is demonstrated that csPDM can accurately capture the firing rate responses in both the steady-state and dynamic regimes over a large range of synaptic time constants from milliseconds to hundreds of milliseconds.

As seen in Equation 24, the resulting quasi-Fokker–Planck equation leaves out all synapse-associated dimensions and just tracks the marginal density function of membrane voltages across the population. As a result, csPDM is extremely computationally efficient even when many types of receptors are included in the network model. The computational speed of csPDM is much faster than that of the original fdPDM by an order of three under the case where one type of receptors is incorporated (Fig. 6). If three types of receptors are incorporated, like in the third example in this study, fdPDM can never be considered as a computationally efficient modeling tool to simulate neural networks because of the inherent difficulty of solving a four-dimensional master equation. Actually, previous studies have argued that solving a master equation with more than three dimensions may spend much more time than MCS (Apfaltrer et al., 2006; Ly and Tranchina, 2007). However, csPDM can efficiently and correctly provides simulations of network activities in this case (Fig. 7). On the other hand, increasing the number of receptor types in the network model does not reduce the accuracy of csPDM considerably. $\eta_{r}^{\text{csPDM}}$ is 0.048 when one type of receptors is included (Fig. 4) and is 0.067 when three types are included (Fig. 7).

When csPDM is compared with MMFM, some interesting findings are observed. First, it is found that MMFM gives quantitatively accurate simulation results only for long synaptic time constants in the steady-state and dynamic regimes (Figs. 3, 4). A possible explanation is that the membrane time constant ($C/g_{l}$) is longer than the synaptic time constant (*τ_s_*) so that the use of the expected value of the steady-state firing rates for estimating the output firing rate (Eq. 18) becomes unreasonable because the neuronal dynamics cannot reach a steady state before synaptic dynamics does. This idea is also evidenced by the decline in $\eta_{r}^{\text{MMFM}}$ when *g_l_* is increased, i.e., reducing the membrane time constant (Fig. 5*B*).

By robustness analysis, we found that the accuracy of MMFM is sensitively influenced by the value of *κ* (Fig. 5*C*), which controls the nonlinearity of EIF neuronal dynamics by adjusting the strength of exponential currents (Eq. 1). Increasing *κ*, i.e., increasing the nonlinearity, decreases the accuracy of MMFM. Actually, MMFM relies on the assumption that the membrane voltage inherits randomness entirely from the stochastic conductance variables, and the conductance variables evolve independently of the membrane voltage (Ly, 2013). However, the author claimed that such an assumption might only be reasonable for linear models such as leaky integrate-and-fire neurons. So, MMFM may not be suitable to simulate network dynamics of nonlinear spiking neurons, like the EIF neurons used in this study. On the contrary, csPDM only assumes that the conductance variables evolve isolatedly, which is true no matter what neuron models are used. Accordingly, it is more general for the type of neuronal models. Furthermore, in the csPDM, the membrane voltage is allowed to be influenced by other random sources, for example, the random release of neurotransmitters (Faisal et al., 2008; Destexhe and Rudolph-Lilith, 2012), which may just add extra noise terms into the stochastic diffusion tensor in the quasi-Fokker–Planck equation without increasing its dimension.csPDM relies on the diffusion approximation of conductance variables. Physiologically, the assumption of diffusion approximation is quite reasonable in that the number of synaptic contacts covering a typical cortical pyramidal cell is indeed very high, in the range of 10^3^–10^4^ (Braitenberg and Schüz, 1991). Each spike at a synaptic contact only alters a post-synaptic potential of <1 mV on average (Markram and Tsodyks, 1996; Markram et al., 1998). So, in our cases, each excitatory or inhibitory spike is designed to cause a maximum change of ≈1 mV in the membrane potential.

Our study is similar to the work of Rudolph and Destexhe (2003), in which the authors employed *Ito*^ *calculus* to formulate the Fokker–Planck equation for Langevin equations and, then, to obtain an analytic expression for the membrane potential distribution at the steady state. They found that steady-state solutions are only available under a limited range of parameter sets. To correct this problem, they used an extended analytic expression for the membrane potential distribution (Rudolph and Destexhe, 2005), by which new solutions became available for a wider range of parameter sets, although the parameters resided in the nonphysiological extreme limits. The major difference of our approach with theirs is the method used to derive the Fokker–Planck equation. In our study, it is based on a modified large-eddy-diffusivity closure method proposed by Barajas-Solano and Tartakovsky (2016), and a quasi-Fokker–Planck equation, i.e., Equation 24, governing the temporal evolution of the membrane potential distribution, is then derived. Although we do not test whether Equation 24 is valid for any parameter sets, by means of our examples in this study, it is found that it at least provides a good solution for parameter sets in physiologic ranges. However, surprisingly, when we apply the same derivation procedures to the neuronal model adopted by them (i.e., Eq. 4.1 in Rudolph and Destexhe, 2005), we obtain the same Fokker–Planck equation as what they derived, in which the diffusion coefficient is also rescaled by $2\tau_{m}\tau_{e}/\left(\tau_{m}+\tau_{e}\right)$ (*τ_m_* is the membrane time constant at rest). This fact implies that Equation 24 might be adequate for an even wider range of parameter sets.

It is theoretically possible to extend our method to any neuron models because no premises are put on the neuronal dynamics. We have tested it on adaptive EIF models (Brette and Gerstner, 2005) and leaky integrate-and-fire-or-burst models (Smith et al., 2000) and find that csPDM works well (data not shown). Such an extension to adaptive EIF neurons is important for the development of PDM in computational neuroscience because the adaptive EIF model has been shown to reproduce a variety of spiking patterns observed in *in vivo* thalamic or cortical neurons (Naud et al., 2008; Touboul and Brette, 2008) and well predict spike times in response to external inputs (Jolivet et al., 2008). csPDM with adaptive EIF models therefore will be a critical research subject in computational neuroscience. We suggest that the synaptic dynamics should be restricted to the first-order kinetics because the calculation of the diffusion tensor, **D** or $D(V,\sigma_{g_{s}})$, is based on an assumption that fluctuation velocity components are exponentially autocorrelated. The calculation becomes more difficult if higher-order synaptic dynamics is used. csPDM only captures STP property of population responses in a qualitative way due to incorrect estimation of $\mu_{x_{s}}$. This poor estimation is caused by a wrong assumption that *u_s_* and *x_s_* are statistically independent for deriving mean-field equations for $\mu_{u_{s}}$ and $\mu_{x_{s}}$ (Tsodyks et al., 1998). In fact, *u_s_* is independent of *x_s_* because *x_s_* does not appear in the Equation 6, but, by contrast, *x_s_* has a negative correlation with *u_s_*. As a consequence, the mean-field equation for $\mu_{x_{s}}$ should include higher-order statistics of both *u_s_* and *x_s_*. However, such an equation is unclosed. Before a closure method is available, the assumption of independence between *u_s_* and *x_s_* is a compromised solution (Tsodyks et al., 1998). Note that it is possible to replace the phenomenological model of STP used here with another elaborating model where state variables are independent with each other. If so, the STP property of population responses can be quantitatively captured by csPDM. This is one of the directions of our future work for improving csPDM.

The derivations of csPDM and numerical examples used for testing are based on a single uncoupled population. Of course, what we derived in this study can be extended to the applications of simulating larger neural networks consisting of multiple coupled populations. However, for the correct use of csPDM, synaptic connections must be sparse, because a large number of synaptic connections can result in the violation of basic assumptions of PDM–Poisson and statistically uncorrelated inputs received by each neuron (Brunel, 2000; Nykamp and Tranchina, 2000; Huertas and Smith, 2006; Augustin et al., 2013). These basic premises can be violated when two neurons share many common pre-synaptic neurons, that is, when there are a huge number of connections between presynaptic and postsynaptic populations. We found that the number of synaptic connections depends on the size of Δ*V_s_*. If Δ*V_s_* is large, the number of connections has to be small because postsynaptic neurons have to receive statistically independent inputs. Postsynaptic neurons are more likely to receive exactly the same inputs if Δ*V_s_* is large and the number of synaptic connections is huge.

## Conclusions

When including many state variables, such as synaptic conductance variables for different types of receptors and synaptic facilitation and depression state variables, the original PDM becomes impractical because of the incredible increase in the computational load. In this study, this critical issue is solved by the probability density function method for Langevin equations with colored driving noise. The newly proposed method, termed csPDM, gives quantitatively accurate firing rate responses in both the steady-state and nonequilibrium regimes and highly qualified estimations of time-varying marginal density functions of membrane voltages. Importantly, csPDM is able to qualitatively manifest STP in an easy way almost without increasing computation demands. It appears that csPDM is generally applicable as a time-saving tool for modeling large-scale neural networks. We hope that our work will inspire further progress in the development of PDM and benefit computational and theoretical studies of synaptic dynamics in network dynamics.

## Synthesis

Reviewing Editor: Gustavo Deco, Universitat Pompeu Fabra Decisions are customarily a result of the Reviewing Editor and the peer reviewers coming together and discussing their recommendations until a consensus is reached. When revisions are invited, a fact-based synthesis statement explaining their decision and outlining what is needed to prepare a revision will be listed below.

Satisfied with all improvements.
